# Multiple early factors anticipate post-acute COVID-19 sequelae

**DOI:** 10.1016/j.cell.2022.01.014

**Published:** 2022-03-03

**Authors:** Yapeng Su, Dan Yuan, Daniel G. Chen, Rachel H. Ng, Kai Wang, Jongchan Choi, Sarah Li, Sunga Hong, Rongyu Zhang, Jingyi Xie, Sergey A. Kornilov, Kelsey Scherler, Ana Jimena Pavlovitch-Bedzyk, Shen Dong, Christopher Lausted, Inyoul Lee, Shannon Fallen, Chengzhen L. Dai, Priyanka Baloni, Brett Smith, Venkata R. Duvvuri, Kristin G. Anderson, Jing Li, Fan Yang, Caroline J. Duncombe, Denise J. McCulloch, Clifford Rostomily, Pamela Troisch, Jing Zhou, Sean Mackay, Quinn DeGottardi, Damon H. May, Ruth Taniguchi, Rachel M. Gittelman, Mark Klinger, Thomas M. Snyder, Ryan Roper, Gladys Wojciechowska, Kim Murray, Rick Edmark, Simon Evans, Lesley Jones, Yong Zhou, Lee Rowen, Rachel Liu, William Chour, Heather A. Algren, William R. Berrington, Julie A. Wallick, Rebecca A. Cochran, Mary E. Micikas, the ISB-Swedish COVID-19 Biobanking Unit, Terri Wrin, Christos J. Petropoulos, Hunter R. Cole, Trevan D. Fischer, Wei Wei, Dave S.B. Hoon, Nathan D. Price, Naeha Subramanian, Joshua A. Hill, Jennifer Hadlock, Andrew T. Magis, Antoni Ribas, Lewis L. Lanier, Scott D. Boyd, Jeffrey A. Bluestone, Helen Chu, Leroy Hood, Raphael Gottardo, Philip D. Greenberg, Mark M. Davis, Jason D. Goldman, James R. Heath

**Affiliations:** 1 Institute for Systems Biology, Seattle, WA 98109, USA; 2 Vaccine and Infectious Disease Division, Fred Hutchinson Cancer Research Center, Seattle, WA 98109, USA; 3 Clinical Research Division, Program in Immunology, Fred Hutchinson Cancer Research Center, Seattle, WA 98109, USA; 4 Department of Bioengineering, University of Washington, Seattle, WA 98105, USA; 5 Department of Microbiology and Department of Informatics, University of Washington, Seattle, WA 98195, USA; 6 Molecular Engineering & Sciences Institute, University of Washington, Seattle, WA 98105, USA; 7 Institute for Immunity, Transplantation and Infection, Stanford University School of Medicine, Stanford, CA 94305, USA; 8 Diabetes Center, University of California, San Francisco, San Francisco, CA 94143, USA; 9 Departments of Immunology and Medicine, University of Washington, Seattle, WA 98109, USA; 10 Department of Pathology, Stanford University, Stanford, CA 94304, USA; 11 Division of Global Health, University of Washington, Seattle, WA 98105, USA; 12 Division of Allergy and Infectious Diseases, Department of Medicine, University of Washington, Seattle, WA 98109, USA; 13 Isoplexis Corporation, Branford, CT 06405, USA; 14 Adaptive Biotechnologies, Seattle, WA 98109, USA; 15 Medical University of Białystok, Białystok 15089, Poland; 16 Swedish Center for Research and Innovation, Swedish Medical Center, Seattle, WA 98109, USA; 17 Providence St. Joseph Health, Renton, WA 98057, USA; 18 Monogram Biosciences, South San Francisco, CA 94080, USA; 19 St. John’s Cancer Institute at Saint John’s Health Center, Santa Monica, CA 90404, USA; 20 Department of Global Heath and Department of Immunology, University of Washington, Seattle, WA 98109, USA; 21 Department of Medicine, University of California, Los Angeles, and Parker Institute for Cancer Immunotherapy, Los Angeles, CA 90095, USA; 22 Department of Microbiology and Immunology, University of California, San Francisco, and Parker Institute for Cancer Immunotherapy, San Francisco, CA 94143, USA; 23 Division of Public Health Sciences, Fred Hutchinson Cancer Research Center, Seattle, WA 98109, USA; 24 Department of Statistics, University of Washington, Seattle, WA 98195, USA; 25 Biomedical Data Sciences, Lausanne University Hospital, University of Lausanne, Lausanne, 1011, Switzerland; 26 Department of Microbiology and Immunology, Stanford University School of Medicine, Stanford, CA 94305, USA; 27 The Howard Hughes Medical Institute, Stanford University School of Medicine, Stanford, CA 94305, USA

**Keywords:** multi-omics, COVID-19, PASC, proteomics, metabolomics, immune system, single-cell TCR-seq, single-cell RNA-seq, single-cell CITE-seq, single-cell secretome, long COVID, symptoms, single cell, transcriptome, computational biology, immunology, viremia, RNAemia, auto-antibodies, antibodies

## Abstract

Post-acute sequelae of COVID-19 (PASC) represent an emerging global crisis. However, quantifiable risk factors for PASC and their biological associations are poorly resolved. We executed a deep multi-omic, longitudinal investigation of 309 COVID-19 patients from initial diagnosis to convalescence (2–3 months later), integrated with clinical data and patient-reported symptoms. We resolved four PASC-anticipating risk factors at the time of initial COVID-19 diagnosis: type 2 diabetes, SARS-CoV-2 RNAemia, Epstein-Barr virus viremia, and specific auto-antibodies. In patients with gastrointestinal PASC, SARS-CoV-2-specific and CMV-specific CD8^+^ T cells exhibited unique dynamics during recovery from COVID-19. Analysis of symptom-associated immunological signatures revealed coordinated immunity polarization into four endotypes, exhibiting divergent acute severity and PASC. We find that immunological associations between PASC factors diminish over time, leading to distinct convalescent immune states. Detectability of most PASC factors at COVID-19 diagnosis emphasizes the importance of early disease measurements for understanding emergent chronic conditions and suggests PASC treatment strategies.

## Highlights

• Longitudinal multi-omics associate PASC with auto-antibodies, viremia, and comorbidities
• Reactivation of latent viruses during initial infection may contribute to PASC
• Subclinical auto-antibodies negatively correlate with anti-SARS-CoV-2 antibodies
• Gastrointestinal PASC uniquely present with post-acute expansion of cytotoxic T cells

## Graphical abstract

**Figure:**
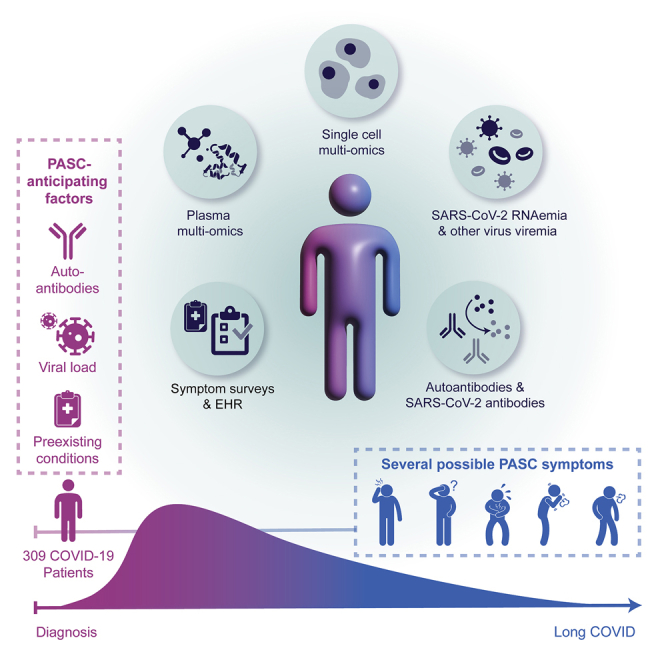

## Teaser

By correlating patient symptoms with in-depth profiling of blood cells and plasma components throughout COVID-19 infection, this study identifies factors that may predict sustained disease.

## Introduction

**Figure 1:**
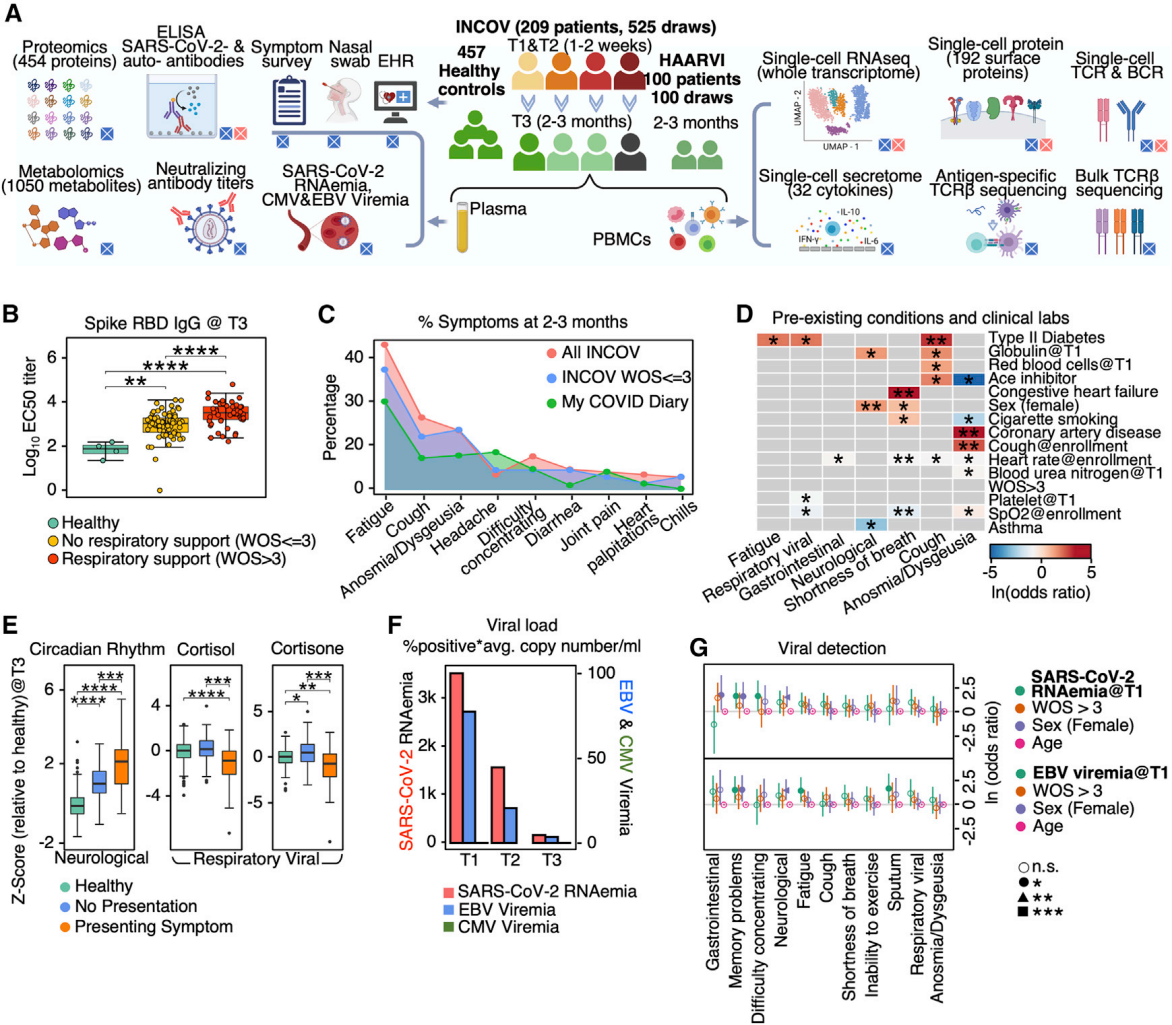
Overview of longitudinal multi-omic analysis of COVID-19 patients and their association with PASC (A) Overview of study design for INCOV and HAARVI cohorts. Assays run on plasma and isolated PBMCs, and patient clinical/symptom data are shown. Bottom-right boxes of each icon denote if assay was performed for INCOV (blue) and/or HAARVI (pink). (B) Boxplots showing ELISA (enzyme-linked immunoassay) measured SARS-CoV-2 RBD IgG antibody titers in healthy individuals and T3 COVID-19 patients with and without respiratory support in their acute stage. ∗∗p value < 0.01, ∗∗∗∗p value < 0.0001. (C) Line plot showing frequency of different symptoms in full INCOV cohort (red), subset of INCOV cohort with acute severity WOS ≤ 3 (no respiratory support), and the MyCOVIDDiary cohort. (D) Heatmap showing the ln(odds ratio) for the associations between pre-existing conditions and clinical measurements from EHR and PASC, adjusted for age, sex, and disease severity (WOS > 3). Associations with significance of p > 0.05 were masked as gray. Only single PASCs that showed statistical significance or the four PASC categories were shown. SpO_2_, blood oxygen saturation. ∗p value < 0.05 and ∗∗p value < 0.01. (E) Boxplots showing plasma protein-based “negative regulation of the circadian rhythm” pathway enrichment (left) and cortisol and cortisone levels (middle and right) from T3 patients with (orange) and without (blue) a specific symptom or from unexposed healthy controls (green). ∗p value < 0.05, ∗∗p value < 0.01, ∗∗∗p value < 0.001, and ∗∗∗∗p value < 0.0001. (F) Barplot showing the viral load level in plasma quantified by the percentage of samples tested positive for viral fragments (RNAemia or viremia) multiplied by the average copy number/mL of these positive samples for SARS-CoV-2 (red), EBV (blue), and CMV (green). (G) Forest plot showing ln(odds ratios) with 95% confidence intervals for associations of PASC with SARS-CoV-2 RNAemia at T1 (top) or EBV Viremia at T1 (bottom), both adjusted for disease severity (WOS > 3, needed respiratory support), sex, and age. The independent associations of disease severity, sex, and age with PASC are also displayed on the same plot. ∗p value < 0.05, ∗∗p value < 0.01, and ∗∗∗p value < 0.001. See also Figure S1 and Tables S1 and S2.

Around 31%–69% of COVID-19 patients suffer from post-acute sequelae of COVID-19 (PASC) (Groff et al., 2021), or long COVID, which is defined (Centers for Disease Control and Prevention, 2021) as a range of new, returning, or ongoing health problems people can experience four or more weeks following initial SARS-CoV-2 infection (Huang et al., 2021; Nalbandian et al., 2021). PASC may include memory loss, gastrointestinal (GI) distress, fatigue, anosmia, shortness of breath, and other symptoms. PASC has been associated with acute disease severity (Blomberg et al., 2021) and is suspected to be related to autoimmune factors (Galeotti and Bayry, 2020) and unresolved viral fragments (Ramakrishnan et al., 2021), although experimental validation on large patient cohorts is still pending. The heterogeneity of PASC and the diverse factors suspected to be associated with it highlight the need to systematically characterize its biological and immunological underpinnings and the evolution of those relationships over the time course of SARS-CoV-2 infection and recovery. To address these knowledge gaps, we carried out a longitudinal multi-omic study of COVID-19 patients (Figure 1A) from initial clinical diagnosis to early-stage recovery from acute disease. We utilized multi-omic systems biology approaches to identify, quantify, and immunologically characterize biological factors associated with and anticipating different PASC.

## Results

### Overview of patient cohorts and PASC

**Figure S1:**
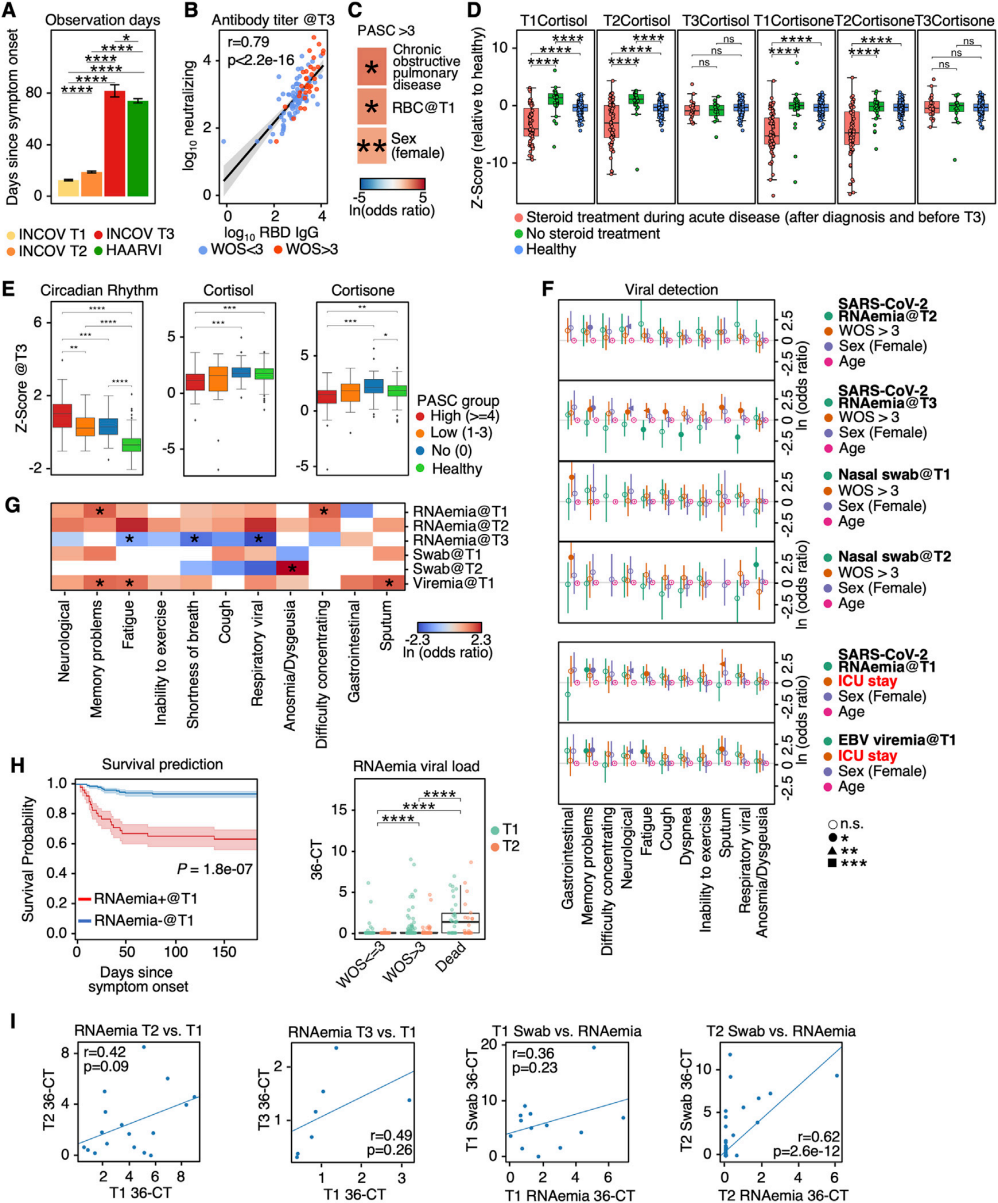
Analysis of antibody titer and modeling for PASC using plasma or swab viral load, related to Figure 1 (A) Barplot showing mean ± SE for the time (days) between symptom onset of COVID-19 to each of the three blood draws for INCOV cohort and the single blood draw for HAARVI cohort. p values calculated from the Mann-Whitney U test are displayed if <0.05. ∗p value < 0.05, ∗∗∗∗p value < 0.0001. (B) Correlation between neutralizing antibody titers at T3 and RBD IgG titers at T3. Data points were fitted with a linear regression line with 95% CI (gray shaded areas), color-coded to indicate whether respiratory support (WOS > 3) was used. Pearson correlation coefficient and p values are shown. (C) Heatmap showing the ln(odds ratio) for only the significant associations between pre-existing conditions and clinical measurements from EHR, and PASC ≥ 4, adjusted for age, sex, and disease severity (WOS > 3). p values calculated from Mann-Whitney U test are displayed if <0.05. ∗p value < 0.05, ∗∗p value < 0.01. (D) Boxplots showing plasma cortisone (left) and cortisol (right) levels at T1, T2, or T3 in patient with and without steroid treatment during COVID-19 infection. p values calculated from the Mann-Whitney U test are displayed if <0.05. ∗p value < 0.05, ∗∗p value < 0.01, ∗∗∗p value < 0.001, and ∗∗∗∗p value < 0.0001. (E) Boxplots showing plasma protein-based “negative regulation of the circadian rhythm” pathway enrichment (left), cortisol (middle), and cortisone (right) levels from healthy individuals (green), T3 patients presenting ≥4 PASC (red), 1–3 PASC (orange), and no PASC (blue). ∗p value < 0.05, ∗∗p value < 0.01, ∗∗∗p value < 0.001, and ∗∗∗∗p value < 0.0001. (F) Forest plot showing ln(odds ratio) with 95% CI for associations between PASCs and variables, including SARS-CoV-2 RNAemia at T2 or T3 (top two panels) and nasal-swab viral loads at T1 and T2 (top five panels), calculated from logistic regression models, with each association/model accounting for disease severity (WOS > 3), sex, and age. Associations between PASC and SARS-CoV-2 RNAemia at T1 or EBV viremia at T1 accounting for ICU stay instead of WOS > 3 are shown (bottom two panels). p values are displayed in asterisks if <0.05. ∗p value < 0.05, ∗∗p value < 0.01, and ∗∗∗p value < 0.001. (G) A summary heatmap showing associations between various SARS-CoV-2 viral load measurements and PASC, accounted for sex, age, and disease severity (WOS > 3). Each rectangle represents the ln(odds ratio) determined through multi-variate logistic regression. p values are displayed if <0.05. ∗p value < 0.05. (H) Left: Kaplan-Meier curves for patient survival stratified by positive (cycle threshold [CT] < 36) or negative for RNAemia at T1. Right: boxplot showing the RNAemia viral load expressed as (36-CT) of patients with different disease severities (WOS ≤ 3, WOS > 3 [not including dead], or dead) at T1 (green) and T2 (orange). ∗∗∗∗p value < 0.0001. (I) Scatter plots fitted with linear regression lines showing correlations between RNAemia measurements at different time point (left two panels), as well as correlations between RNAemia and nasal-swab viral loads (right two panels). Pearson correlation coefficients and p values are displayed.

Our primary cohort (INCOV) of 209 patients represented the spectrum of acute infection severities (Tables 1 and S1.1) and was paired with 457 healthy controls (Table S1.2). These patients were studied at clinical diagnosis (T1), acute disease (acute, T2), and 2–3 months post onset of initial symptoms (convalescent, T3) (Figures 1A and S1A). Blood draws were analyzed for auto-antibodies (autoAbs) and SARS-CoV-2-specific antibodies, global plasma proteomic and metabolomic profiles, and single-cell (sc) multi-omic characterizations of peripheral blood mononuclear cells (PBMCs). Each blood draw was paired with nasal-swab and plasma measurements of SARS-CoV-2 viral load. These datasets were integrated within the context of electronic health records (EHRs) and self-reported symptoms of the same patients to guide the interpretation of the molecular signatures of PASC within a clinical context (Figure 1A). We performed a subset of analyses on an independent cohort of 100 post-acute COVID-19 patients (hospitalized or ambulatory adults with respiratory-viral infections [HAARVI] cohort) to validate the findings from our primary cohort (Figure 1A; Tables 1 and S1.4). The duration between symptom onset and the draw of the HAARVI cohort was nearly identical to that of the T3 draw of INCOV (Figure S1A).

**Table 1:** Demographics and clinical characteristics

	INCOV individuals (n = 209)	HAARVI individuals (n = 100)	Healthy individuals (n = 457)
	Blood draw (n = 525)	Blood draw (n = 100)	
**Demographics**			

Age in years, mean ± SD (range)	56 ± 18 (18–89)	50 ± 15 (23–76)	49 ± 12 (19–80)
Female	50% (104/209)	66% (66/100)	60% (272/457)
BMI, mean ± SD (range)	30 ± 7 (14–56)	27 ± 6 (18–55)	28 ± 6 (17–53)
Ethnicity: hispanic or latino	15% (32/209)	3% (3/100)	N/A
Ethnicity: not hispanic	81% (169/209)	96% (96/100)	N/A
Race: White	51% (106/209)	91% (91/100)	N/A
Race: Asian	13% (28/209)	8% (8/100)	N/A
Race: Black or African American	10% (21/209)	3% (3/100)	N/A
Race: Native Hawaiian or other Pacific Islander	3% (6/209)	N/A	N/A
Race: American Indian/Alaska native	1% (3/209)	3% (3/100)	N/A
Race: more than one race	1% (2/209)	7% (7/100)	N/A

**Clinical characteristics**			

Hospital admission	71% (148/209)	10% (10/100)	N/A
Respiratory support	56% (118/209)	N/A	N/A
ICU admission	30% (62/209)	5% (5/100)	N/A
Intubation and mechanical ventilation	18% (38/209)	3% (3/100)	N/A

**Comorbidities**			

Hypertension	40% (84/209)	14% (14/100)	N/A
Diabetes	23% (47/209)	6% (6/100)	N/A
Type 1 diabetes	1% (2/209)	N/A	N/A
Type 2 diabetes	22% (45/209)	N/A	N/A
Asthma	16% (33/209)	1% (1/100)	N/A
Chronic obstructive pulmonary disease	6% (13/209)	2% (2/100)	N/A
Cardiovascular disease	N/A	2% (2/100)	N/A
Congestive heart failure	7% (14/209)	N/A	N/A
Coronary artery disease	8% (16/209)	N/A	N/A
Cancer	11% (23/209)	N/A	N/A
Chronic kidney disease	8% (17/209)	N/A	N/A
Immunocompromised	4% (9/209)	N/A	N/A
HIV infection	0% (0/209)	1% (1/100)	N/A

Numerical variables were shown in mean ± σ (minimum to maximum). Categorical variables were shown in percentages (number of the observation/total number of patients). Not all data were collected for healthy individuals. ICU, intensive care unit; COPD, chronic obstructive pulmonary disease; HIV, human immunodeficiency virus. Detailed information can be found in Tables S1.1–S1.4.

At T3, most participants exhibited antibodies against the SARS-CoV-2 spike protein receptor-binding domain (RBD) (Figure 1B). Antibody titers correlated with acute disease severity, as expected (Röltgen and Boyd, 2021), and also with neutralizing antibodies in cell-based assays (Figure S1B), suggesting that most patients exhibited robust antibody responses against SARS-CoV-2 by T3.

Patient-reported symptoms from interviews were validated and confirmed to be COVID-19-related through EHR. At T3, symptoms included fatigue (52% of participants), cough (25%), and anosmia/dysguesia (18%) (Table S1.3). Some specific PASC may be reported by only a small fraction of our cohort. Thus, we also classified symptoms as respiratory viral (42%), neurological (25%), anosmia/dysgeusia (18%), and GI (9%) (Table S1.3). Studies on PASC have used heterogeneous inclusion criteria, symptom definitions, and observation windows but show a pattern where respiratory-viral symptoms are more common and GI symptoms are rarer (Groff et al., 2021; Jiang et al., 2021). Both the INCOV cohort and a separate cohort, MyCOVIDDiary (Providence, 2021), showed similar trends (Figure 1C), suggesting that the symptoms reported by the INCOV cohort are reasonably representative. Interestingly, patients with mild and severe acute COVID-19 severity also exhibited similar trends (Figure 1C), implying that factors beyond acute-stage disease severity could be associated with PASC. T3 seronegative patients (8%) were enriched for immuno-compromised patients and exhibited similar risks of PASC (Table S1.5).

Examination of PASC in the context of EHR data from the INCOV participants revealed significant correlations between type 2 diabetes and certain PASC (Figure 1D). Female patients, patients with chronic obstructive pulmonary disease (COPD), and those with higher T1 RBC counts were more likely to present with many (>3) symptoms (Figure S1C).

### Plasma proteomic and metabolic biomarkers at convalescence associated with PASC

We investigated global plasma proteomic and metabolomic profiles to identify T3 plasma markers associated with different PASC (Tables S2.1 and S2.2). For example, patients reporting respiratory-viral symptoms at T3 exhibited significantly repressed levels of cortisol and cortisone at T3 (Figure 1E). Low cortisol, a glucocorticoid, is the hallmark of adrenal insufficiency (Puar et al., 2016), which is a treatable condition that can cause symptoms reminiscent of many PASC. Low cortisol has been reported in acute COVID-19 patients (Choy, 2020), but not at convalescence. Suppression of endogenous cortisol production could be caused by steroid treatments, as certain steroids are structurally similar to cortisol and may cause feedback inhibition of cortisol production (Younes and Younes, 2017). We did observe a significant association between steroid treatment and cortisol/cortisone levels at the times of T1 and T2 but not at T3 (Figure S1D). Additionally, patients reporting neurological symptoms exhibited elevated proteins associated with the negative regulation of the circadian sleep/wake cycle (Figure 1E). Interestingly, both the cortisol downregulation and circadian rhythm elevation are further enriched in patients with many (>3) symptoms at T3 (Figure S1E). These biomarkers may help clinically define PASC and suggest distinct origins of PASC subsets. This prompted us to conduct a deeper multi-omic characterization of their etiology.

### Latent EBV reactivation and SARS-CoV-2 RNAemia at COVID-19 diagnosis anticipate PASC

Reactivation of Epstein-Barr virus (EBV) has been indirectly inferred to correlate with PASC through antibody titer measurements (Gold et al., 2021). We directly probed for the reactivation of latent viruses by measuring plasma viremia of EBV and a second common latent virus, cytomegalovirus (CMV). We also probed for circulating mRNA fragments of SARS-CoV-2 (RNAemia) (Figure 1F; STAR Methods). We detected EBV viremia at T1 in 14% of tested patients, and positive SARS-CoV-2 RNAemia in 25% of patients, with few individuals positive for both (Table S2.3). For both viral assays, signals dropped 2- to 3-fold between T1 and T2 and were barely detected at T3 (Figure 1F). CMV viremia was not detected.

We analyzed whether EBV viremia (at T1), SARS-CoV-2 RNAemia (at T1), or SARS-CoV-2 viral load from nasal swabs was significantly associated with PASC at T3 (Figures 1G, S1F, and S1G). For these analyses, we corrected for contributions from age, sex, and acute COVID-19 severity. COVID-19 severity was defined as whether respiratory support was needed, or by correcting for intensive care unit (ICU) admission (Figure S1F).

Although memory PASC was significantly associated with T1 measures of both EBV viremia and SARS-CoV-2 RNAemia, the fatigue and sputum PASC were specific to EBV viremia (Figure 1G). Very few patients exhibited positive EBV viremia at T2 or T3 or positive SARS-CoV-2 nasal-swab viral loads at T3 to facilitate their analysis. T1 SARS-CoV-2 RNAemia also provided a biomarker of mortality (Figure S1H) as reported in Gutmann et al. (2021). SARS-CoV-2 nasal-swab viral load significantly associated only with anosmia/dysgeusia (Figures S1F and S1G) and only at T2. RNAemia associations between different time points and with nasal swabs showed weak correlations (Figure S1I). In summary, reactivation of latent EBV and SARS-CoV-2 RNAemia at T1 are factors that anticipate, to varying degrees, PASC at T3.

### Auto-antibodies anticorrelate with anti-SARS-CoV-2 antibodies and are associated with distinct patterns of PASC

**Figure S2:**
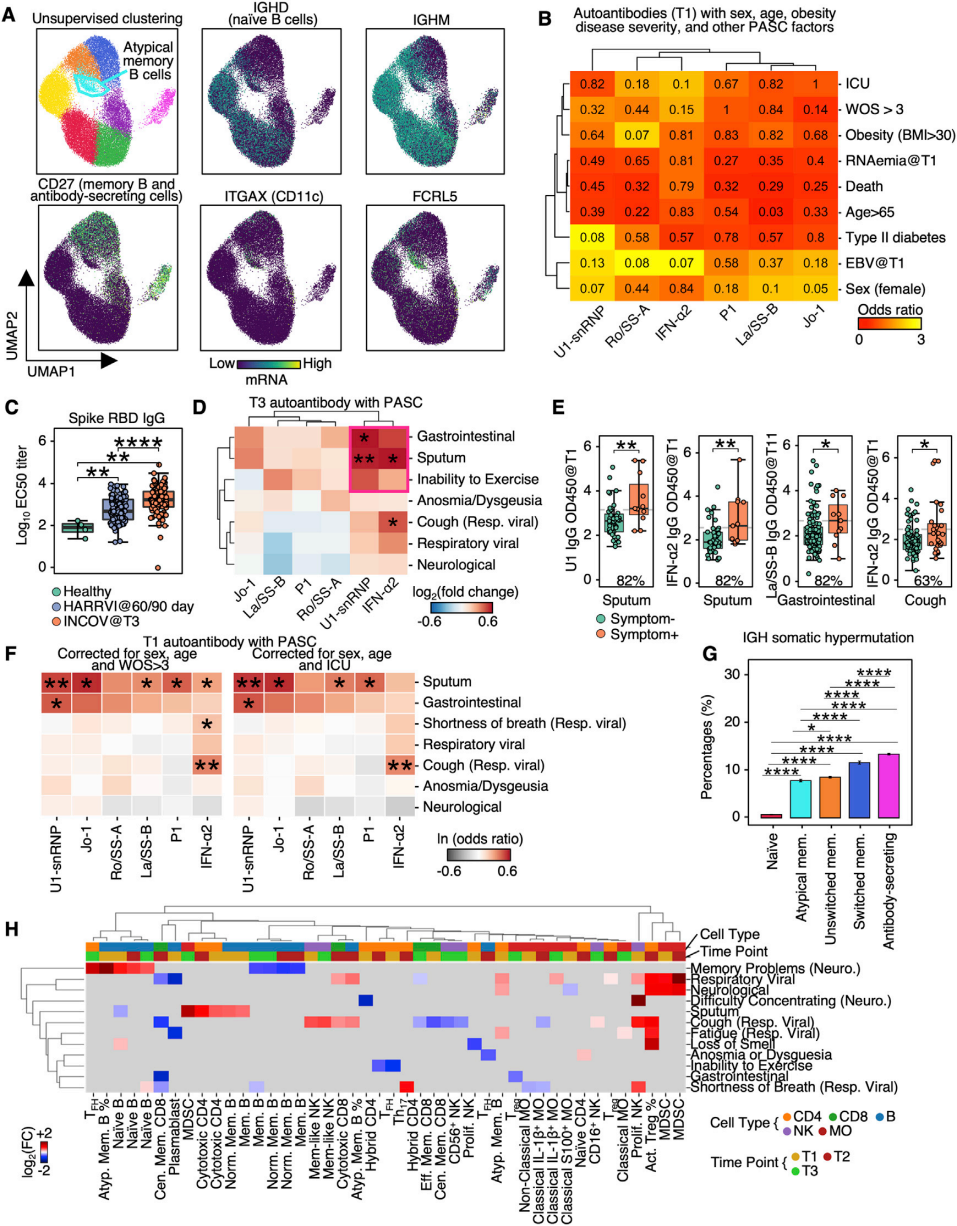
Auto-antibodies anticorrelate with anti-SARS-CoV-2 antibodies and are associated with distinct patterns of PASC, related to Figure 2 (A) UMAP visualization of single B cells color-coded by Leiden clusters (top left) and selected gene transcript levels (other panels). (B) Heatmap showing the odds ratio (color-coded) and p values (shown in numbers) from Fisher’s exact test to determine the dependence of column and row variables. (C) Boxplot showing titers of SARS-CoV-2 spike RBD antibodies in healthy, INCOV, and HAARVI at convalescence (T3 for INCOV, and 2–3 months post infection for HAARVI). p values calculated from the Mann-Whitney U test are displayed. ∗∗p value < 0.01, and ∗∗∗∗p value < 0.0001 (D) Hierarchical clustered heatmap showing log_2_-fold change of T3 autoantibody levels in patients with a specific PASC (rows) compared to those without. p values calculated from the Mann-Whitney U test are displayed if <0.05. ∗p value < 0.05, ∗∗p value < 0.01. (E) Boxplots showing all significant PASC- autoantibody (T1) relationships in Figure 2C. The percentages of patients with a given PASC that had autoantibody levels greater than the median antibody level of those who did not present the PASC are shown. p values calculated from the Mann-Whitney U test are displayed if <0.05. ∗p value < 0.05, ∗∗p value < 0.01. (F) Heatmap showing associations between T1 autoantibody measurements and PASC, accounted for sex, age, and disease severity (left: WOS > 3, right: ICU). Each rectangle represents the ln(odds ratio) determined through multi-variate logistic regression. p values are displayed if <0.05. ∗p value < 0.05, ∗∗p value < 0.01. (G) Bar plots showing mean ± SE somatic hypermutation rates in CDR regions of the heavy chain in different B cell populations. p values calculated from Mann-Whitney U test then corrected as FDR via the Benjamini-Hochberg method are displayed if FDR < 0.05. ∗FDR < 0.05, ∗∗∗∗FDR < 0.0001. (H) Associations between phenotype percentages as measured for all three time points (columns) and PASC (rows). The immune cell class is color-coded on the top row, and the measurement time point is color coded on the second row. Enrichment is quantified as log_2_-fold changes between patients with PASC compared with those without. These are colored as red for positive, blue for negative, and statistically non-significant fold changes are shown as gray (p ≥ 0.05).

AutoAbs, especially those that neutralize type I interferons (IFNs), have been reported to be associated with immune dysfunction and COVID-19 mortality (Bastard et al., 2021; Wang et al., 2021) and have been speculated to be associated with PASC (Proal and VanElzakker, 2021). We investigated the possibility for such a link by measuring a panel of autoAbs at T1 and T3 and comparing them against anti-SARS-CoV-2 Abs of different isotypes. The autoAb panel included anti-IFN-α2, and five anti-nuclear autoAbs (ANAs) (Ro/SS-A, La/SS-B, U1-snRNP, Jo-1, and P1) commonly associated with systemic lupus erythematosus (SLE) (Choi et al., 2020; Pisetsky and Lipsky, 2020). SLE is an autoimmune disease that shares certain symptoms with PASC (Raveendran et al., 2021) and has also been reported to manifest following COVID-19 (Zamani et al., 2021). The SLE-associated ANAs have already been detected in acutely infected COVID-19 patients (Chang et al., 2021). The use of the SLE-ANA-panel was additionally supported by the observed expansion of atypical memory B cells (AtMs, *IGHD*^*−*^CD27^−^CD11c^+^*FCRL5*^+^ [defined in Figure S2A]) in both COVID-19 and SLE patients (Oliviero et al., 2020; Su et al., 2020). In SLE, AtMs are generated during chronic inflammation, enriched with autoreactivities, and correlated with disease activities (Jenks et al., 2018).

**Figure 2:**
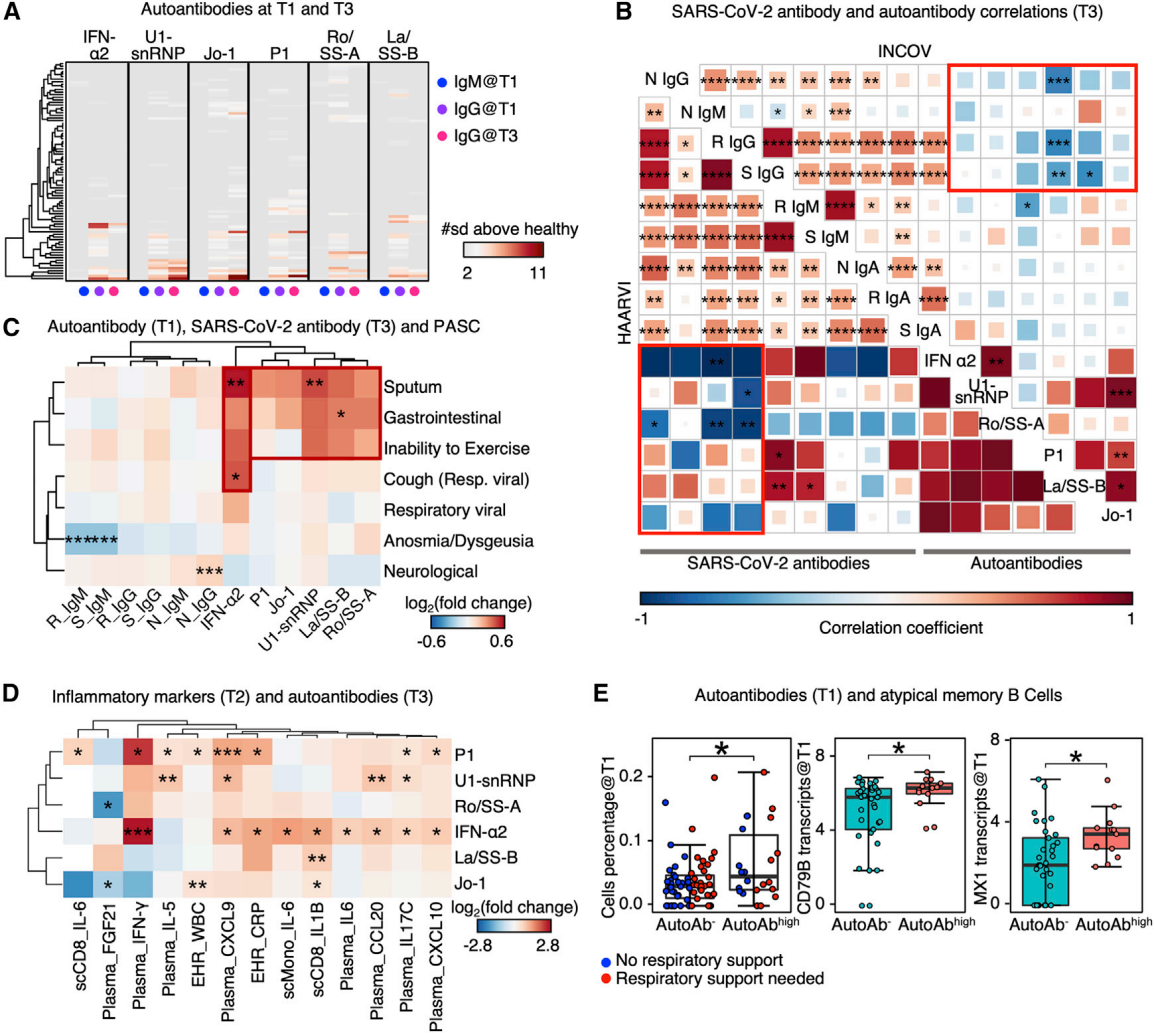
Auto-antibodies anticorrelate with anti-SARS-CoV-2 antibodies and are associated with distinct patterns of PASC (A) Heatmap showing the IgM at T1, IgG at T1, and IgG at T3 for each autoantibody annotated at the top. Each row represents a patient. Only patients with measured autoantibody levels above 2 standard deviations (σ) of healthy individuals are shown. (B) Two aligned correlation matrices assembled from INCOV (upper right) and HAARVI cohorts (lower left). Each square represents the correlation coefficient between an antibody pair specified by the diagonal annotations. p values of these correlations are displayed in asterisks if <0.05. ∗p value < 0.05, ∗∗p value < 0.01, ∗∗∗p value < 0.001, and ∗∗∗∗p value < 0.0001. N, nucleocapsid protein; S, spike protein; R, RBD domain of spike; Ig, immunoglobin. Pink rectangles highlight the overall anti-correlation trends between auto-antibodies and anti-SARS-CoV-2 IgGs. (C) Hierarchical clustered heatmap showing log_2_-fold change of T3 SARS-CoV-2 antibody or T1 autoantibody levels in patients with a specific PASC (rows) compared with those without. p values calculated from the Mann-Whitney U test are displayed if <0.05. Only single PASCs that showed statistical significance or the four PASC categories were shown. ∗p value < 0.05, ∗∗p value < 0.01, and ∗∗∗p value < 0.001. (D) Hierarchical clustered heatmap showing log_2_-fold change of EHR clinical labs, plasma analytes, or transcript levels in immune cells (annotated within column names), in patients with auto-antibodies (>2σ + healthy) to those without (≤2σ + healthy). p values calculated from the Mann-Whitney U test are displayed in asterisks if <0.05. ∗p value < 0.05, ∗∗p value < 0.01, and ∗∗∗p value < 0.001. (E) Boxplots showing the cell percentage (left), *CD79B* transcript levels (middle), and *MX1* transcript levels (right) of atypical memory B cells in patients without any auto-antibodies (autoAb^−^, ≤2σ + healthy) and those had any autoantibody levels ≥4σ + healthy (autoAb^high^). p values calculated from the Mann-Whitney U test are displayed in asterisks if <0.05. ∗p value < 0.05. See also Figure S2 and Tables S2 and S5.

We had several major findings. First, we observed that patients with autoAbs at T3 (44%) already exhibited mature (class-switched) autoAbs as early as at diagnosis (56%) (Figure 2A), indicating that the autoAbs may predate COVID-19, as reported elsewhere (Bastard et al., 2021). Analysis of EHR data confirmed that only 6% of autoAb-positive patients had documented autoimmune conditions before COVID-19, suggesting that the autoAbs may reflect subclinical conditions.

Second, we found interesting cross-correlations between autoAbs and anti-SARS-CoV-2 Abs at T3 (Figure 2B). Anti-SARS-CoV-2 IgG titers positively correlated with each other, as did the autoAbs. However, all significant correlations between SARS-CoV-2 IgGs (class-switched) and autoAbs (anti-IFN-α2 and anti-nuclear) are anticorrelations. These findings were validated through the independent HAARVI cohort (Figure 2B, pink rectangles). Notably, the HAARVI participants experienced mild COVID-19 relative to the INCOV participants (10% versus 71% hospitalization rates, Tables 1, S1.1, and S1.4) and therefore had lower levels of anti-SARS-CoV-2 antibodies (Figure S2C), potentially explaining why some specific correlations do not track across the two cohorts, although the overall trends do hold.

A third major finding was that anti-SARS-CoV-2 Abs and specific autoAbs were associated with different PASC. For example, patients with neurological PASC exhibited slightly higher levels of anti-SARS-CoV-2 nucleocapsid protein IgG, whereas GI-related PASC and sputum production were associated with elevated levels of multiple autoAbs at T3 (Figure S2D; Table S2.4) and even T1 (Figures 2C and S2E; Table S2.4). IFN-α2 autoAbs uniquely associated with respiratory-viral PASC, even after correcting for age, sex, and disease severity (Figures 2C, S2B, and S2F; Table S2.4). These observations suggest that T1 autoAb levels may be anticipating biomarkers of certain PASC (Figures 2C and S2F).

The negative correlations between anti-SARS-CoV-2 Abs and autoAbs suggest two lines of inquiry. First, anti-IFN-α2 may neutralize IFN-α2 signaling, dysregulating IFN-dependent B cell responses (Braun et al., 2002), and limiting virus-specific Ab production. IFN-α2 inhibition may also upregulate pro-inflammatory cytokines (Guarda et al., 2011), promoting ANA generation against self-antigens from tissue damage (Smatti et al., 2019). Consistently, we found in T2 (acute stage) plasma that multiple inflammation biomarkers, including IFN-γ, C-reactive protein, and IL-6, were positively associated with autoAbs at T3 (Figure 2D; Table S2.4). Similarly, in monocytes and CD8^+^ T cells at T2, these autoAb-positive patients exhibited upregulated expression of pro-inflammatory cytokine genes (Figure 2D). The consistency across data modalities suggests a notable connection between autoAbs, T2 hyperinflammation and T3 PASC.

A second line of inquiry involved the AtM B cells, which have been shown to be precursors of autoAb-producing plasma cells in SLE (Jenks et al., 2018). AtMs originate from extrafollicular pathway activation of both naive and memory B cells (Sokal et al., 2021) and exhibit lower levels of somatic hypermutation (SHM) than other memory B cells, consistent with our data (Figure S2G). The upregulation of AtMs in COVID-19 was most pronounced in high-autoAb patients (Figure 2E, left). Furthermore, in these patients, upregulated expression within AtMs of the B cell receptor (BCR) signaling molecule *CD79B* and the IFN-inducible gene *MX1* (Figure 2E, middle and right; Table S2.5) implied enhanced BCR and IFN signaling (Michalska et al., 2018), reminiscent of the hyperactive state of B cells seen in SLE (Domeier et al., 2018), and associated with the over-production of lupus autoAbs (Jenks et al., 2018). This analysis suggests a potential SLE-shared mechanism for the generation of autoAbs.

### Unique T cell clonal dynamics reveal distinct GI PASC associations

**Figure 3:**
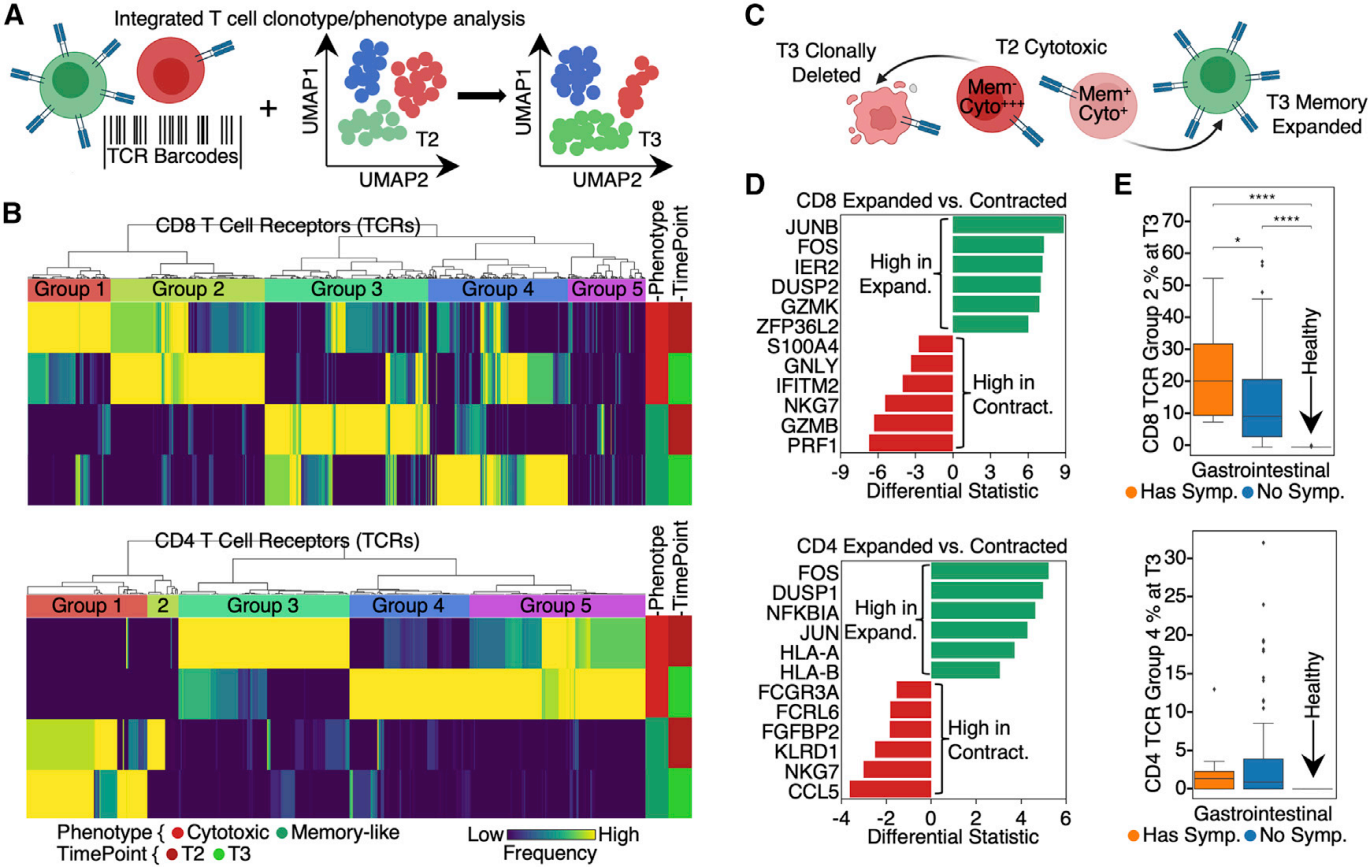
Lineage tracing of T cell clonotypes along the transcriptomic landscape resolved PASC association with global clonal and transcriptomic dynamics (A) Illustration of using TCRs as T cell lineage barcodes to trace how different clonotypes evolve along transcriptomic landscape from acute disease (T2) to convalescence. (B) Hierarchical clustering of CD8^+^ (upper panel) and CD4^+^ T cell (lower panel) TCRs (columns) based on TCR sharing patterns across select phenotypes and time points (see color key at bottom). (C) Illustration of mining differential transcriptomic features for CD8^+^ and CD4^+^ T cells that are of a cytotoxic T_EMRA_ phenotype at T2 but expand into a memory phenotype at T3, or contract at T3. (D) Top differentially expressed genes at T2 between cytotoxic T_EMRA_ cells that either expand into a memory phenotype, or contract by T3. CD8^+^ (top panel) and CD4^+^ T cells (bottom panel). (E) Frequencies of newly emerging cytotoxic clonotypes (TCR group 2 for CD8^+^ T cells in (B) top heatmap, TCR group 4 for CD4^+^ T cells in (B) bottom heatmap) for patients at T3 with (orange) and without (blue) GI symptoms and for unexposed healthy controls (green). p values calculated from the Mann-Whitney U test are displayed in asterisks if <0.05. ∗p value < 0.05, ∗∗p value < 0.01, ∗∗∗p value < 0.001, and ∗∗∗∗p value < 0.0001. See also Table S3.

T cell clonal dynamics, as inferred from T cell receptor (TCR) gene sequences and sc transcriptomics, can provide insights into the evolution of the adaptive immune response over the course of infection and recovery. We used TCR genes as barcodes to track the T2 to T3 dynamics of CD8^+^ and CD4^+^ T cell clonotypes (Figure 3A). This analysis revealed that, for both phenotypes, the clonally dominant TCRs at T3 are different from those at T2 (Figure 3B). For example, in CD8^+^ T cells, TCR groups 1 and 2 were both enriched for the cytotoxic T_EMRA_-like phenotype. However, group 1 TCRs were dominant at T2 and contracted at T3, whereas group 2 TCRs were dominant at T3 but not T2 (Figure 3B, upper; Table S3.5). Similar dynamics were seen for CD4^+^ T cells (Figure 3B, lower, Table S3.6).

Inspired by these divergent clonal-transcriptomic dynamics, we queried for early (T2) transcriptional differences between cytotoxic T_EMRA_-like CD8^+^ T cells that transitioned to effector memory (EM) T cells at T3 (group 4) versus those that clonally contracted (group 1) (Figure 3C). The “memory-precursor” clonotypes showed biased upregulation of genes that inhibit inflammation or prevent T cell over-activation (e.g., *DUSP2* [Lang and Raffi, 2019] and *JUNB* [Koizumi et al., 2018]) (Figure 3D, upper; Table S3.1). By contrast, the effector clonotypes destined for contraction had upregulated genes associated with effector functions (e.g., *GZMB* and *PRF1*) and inflammatory responses (Figure 3D, upper; Table S3.2). Similar signatures were also observed for CD4^+^ T cells (Figure 3D, lower; Tables S3.3 and S3.4). The implication is that for cytotoxic T cell phenotypes, differences in early transcriptional programs may lead to divergent cell fates. These observed behaviors of T cell clonal contraction and memory-formation likely reflect normal recovery from COVID-19, similar to those in other viral infection settings (Kaech et al., 2002).

However, counterintuitively, we note that the pool of cytotoxic T cells is also replenished with newly expanded clonotypes even at T3 (Figure 3B; CD8 group 2 and CD4 group 4), perhaps suggesting an unusual recovery for some patients. Furthermore, this expanded cytotoxic pool was significantly enriched in patients reporting GI PASC (Figure 3E, upper; Table S3.7). Similarly, newly emerging cytotoxic CD4^+^ T cells (group 4) at T3 appeared enriched in GI PASC-positive patients (Figure 3E, lower; Table S3.8). These analyses suggest that GI PASC is associated with unique T cell clonal and transcriptome dynamics, prompting us to explore the antigen specificity of these T cell populations.

### Different activation dynamics of SARS-CoV-2-specific T cell are associated with distinct PASC

**Figure 4:**
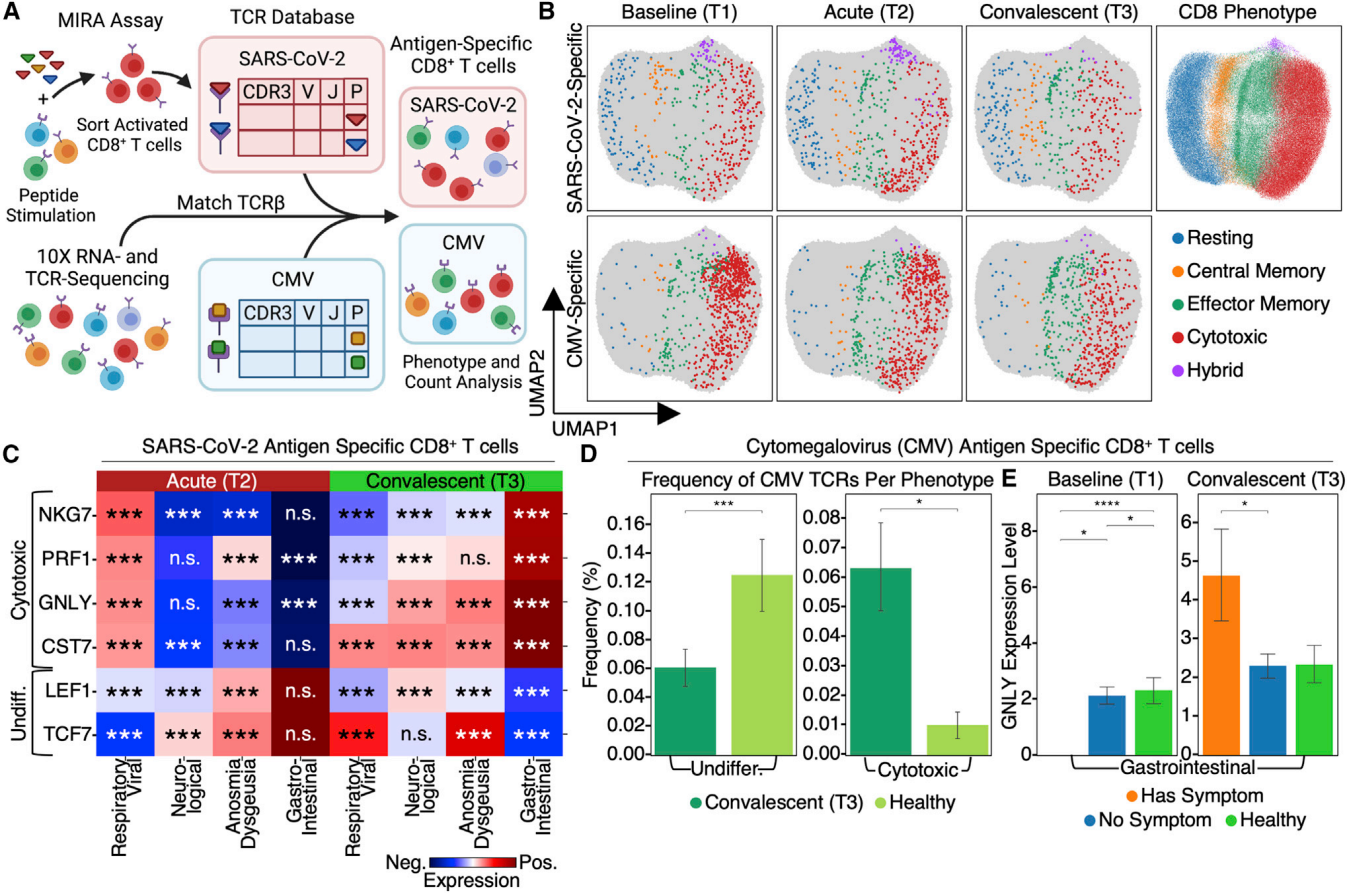
Integration of antigen specificity with sc-CITE-seq data reveal PASC associations with SARS-CoV-2-specific and CMV-specific TCR-transcriptomic dynamics (A) Illustration of the computational pipeline that integrates SARS-CoV-2-specific TCRs from the MIRA analysis and CMV-specific TCRs from public databases, with CD8^+^ T cell transcriptomes from sc-CITE-seq data. (B) UMAP (Uniform Manifold Approximation and Projection) visualization of transcriptomic states of SARS-CoV-2-specific T cells and CMV-specific T cells from T1 through T3. (C) Heatmaps showing select mRNA enrichment in SARS-CoV-2-specific CD8^+^ T cells for patients with certain PASCs compared with those without. p values calculated from the Mann-Whitney U test are displayed in asterisks if <0.05. ∗p value < 0.05, ∗∗p value < 0.01, and ∗∗∗p value < 0.001. (D) Frequency of CMV-specific undifferentiated and cytotoxic CD8^+^ T cells in patients at T3 (dark green) in comparison with unexposed healthy controls (light green). Data are represented as mean ± SE. p values calculated from the Mann-Whitney U test are displayed in asterisks if <0.05. ∗p value < 0.05, ∗∗p value < 0.01, and ∗∗∗p value < 0.001. (E) mRNA levels of *GNLY* in CMV-specific CD8^+^ T cells in patients at T1 and T3 with (orange) and without (blue) GI symptoms in comparison with unexposed healthy controls (green). Data are represented as mean ± SE. p values calculated from the Mann-Whitney U test are displayed in asterisks if <0.05. ∗p value < 0.05, ∗∗p value < 0.01, ∗∗∗p value < 0.001, and ∗∗∗∗p value < 0.0001. See also Table S4.

To investigate the transcriptional dynamics of SARS-CoV-2-specific T cells, we first performed a functional assay for the multiplex identification of TCR antigen specificities (MIRA) (Snyder et al., 2020) on COVID-19 patient PBMCs to identify over 150,000 TCRs specific to nearly 600 epitopes spanning the entire SARS-CoV-2 viral proteome (Figure 4A; Table S4.1). These functional TCRs were integrated with sc-CITE-seq (single-cell Cellular Indexing of Transcriptomes and Epitopes by Sequencing) data (Figure 4A) to reveal the transcriptome of SARS-CoV-2-specific CD8^+^ T cells (Figure 4B, upper; Table S4.3). For patients reporting GI PASC, SARS-CoV-2-specific CD8^+^ T cells exhibited undifferentiated phenotypes during acute disease and elevated cytotoxic characteristics at T3 (Figure 4C). By contrast, in patients with respiratory-viral symptoms, SARS-CoV-2-specific T cells followed the opposite trend (Figure 4C; Table S4.5). These divergent dynamics for different symptoms suggest that GI PASC and respiratory-viral PASC may have different origins.

### CMV-specific T cell bystander activation associates with GI PASC

Bystander activation describes the case when T lymphocytes with specificities for unrelated epitopes are activated during an antigen-specific response (Whiteside et al., 2018). We queried its potential association with PASC by isolating T cells specific for CMV but not SARS-CoV-2 (Figure 4B, lower; Table S4.2; see STAR Methods). Interestingly, CMV-specific CD8^+^ T cells from COVID-19 patients displayed distinct transcriptome characteristics relative to unexposed healthy controls, with more cytotoxic and less naive-like signatures even at T3 (Figure 4D). Notably, although the absolute numbers of cytotoxic CMV-specific CD8^+^ T cells decrease from T1 to T3, those cells that do persist at T3 are positively associated with GI PASC (Figure 4E; Tables S4.4 and S4.6), similar to what was found for SARS-CoV-2-specific T cells. *GNLY* was utilized as a surrogate marker and showed the same trend as that of other cytotoxic markers, such as *GZMB* and *PRF1* (Table S4.6). These observations, coupled with the absence of detectable CMV viremia, suggest an association of bystander activation of CMV-specific CD8^+^ T cells with GI PASC.

### Unresolved dysregulated immune phenotypes associate with different PASC

Immune dysregulation has been suspected to be associated with PASC (Proal and VanElzakker, 2021), although experimental evidence remains elusive. We probed for global immunological signatures of PASC by first analyzing the sc transcriptomes of over 1,000,000 PBMCs collected from all samples in the INCOV cohort. Cells were classified into major immune cell types and subtypes based on global transcriptomic profiles (see STAR Methods). Interestingly, many immune cell phenotypes reported to be associated with severe acute COVID-19 remain enriched at T3, to varying degrees, and to associated with PASC. These include cytotoxic CD4^+^ T cells, proliferative-exhausted (hybrid) T cells and myeloid-derived suppressor cells (MDSCs) (Lee et al., 2022; Mathew et al., 2020; Schulte-Schrepping et al., 2020; Su et al., 2020; Zheng et al., 2021) (Tables S5.1–S5.5). For example, MDSCs, which can indicate immune paralysis and serve as a predictor for mortality in acute COVID-19 (Su et al., 2020), remain upregulated at T3 in patients with sputum PASC (Figure S2H). Similarly, a memory-like NK cell subtype at T3 positively associates with cough PASC (Figure S2H). Interestingly, activated T_reg_ at T2 significantly positively anticipates many different PASCs (Figure S2H). Analysis of how sc transcriptomes change over time also revealed that the innate immunity arm may exhibit persistent activation at T3 via NF-kB activation mediated by TRAF6 (Table S5.6).

### Systematic association of PASC and immune transcriptomes reveals four immune endotypes

**Figure 5:**
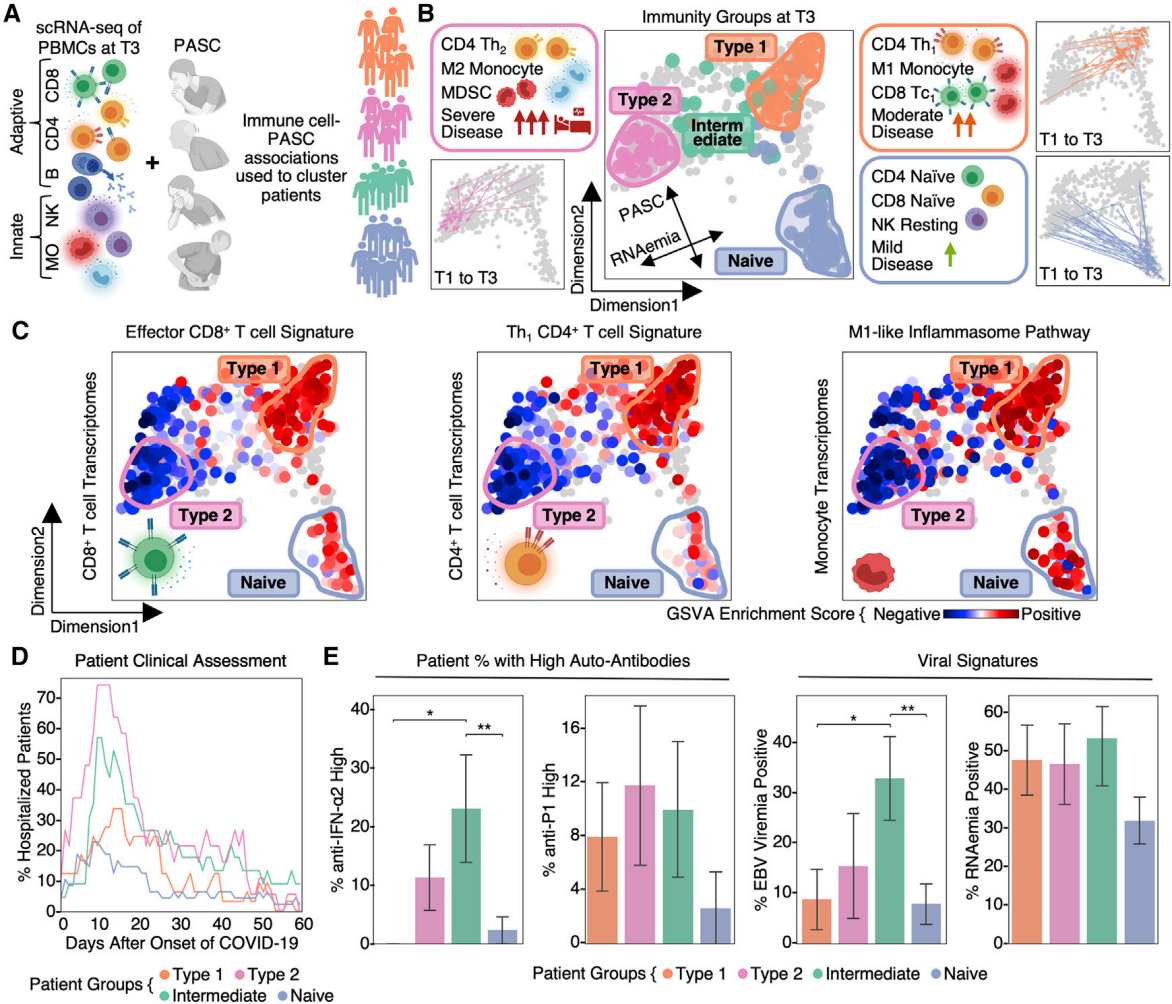
Global immunological association of PASC revealed coordinate polarization of innate and adaptive immunity into four immune endotypes (A) Illustration of the computational pipeline that integrates the immune transcriptomes for each cell type with PASC and uses this integration to classify and place patients on a low-dimensional projection. (B) Two-dimensional projection of immune-symptom signatures. Each dot represents a patient blood draw, increased distance between dots represents increased dissimilarities. Identified patient groups in (A) are color-coded on T3 blood draws. Representative characteristics are summarized in the side boxes. Trajectories connecting the T1 and T3 patient blood draws for three of the groups are shown at the side. (C) Pathway analysis of patient-group-specific transcriptomic signatures for CD8^+^, CD4^+^ T cell, and monocytes across patients. Enrichment scores of selected pathways in CD8^+^, CD4^+^ T cells, and monocytes for each blood draw are color coded onto each dot. (D) Real-time hospitalization rates for each of the four patient endotype. (E) Left: percent of patients per immunity endotype that had high IFN-α2 or P1 auto-antibodies at T1 (defined as ≥4 standard deviations above healthy controls) when considering autoAb^high^ and autoAb^−^ patients. Right: percent of patients with EBV viremia or SARS-CoV-2 RNAemia levels that cross the threshold for positivity. Data are represented as mean ± SE. p values calculated from the Mann-Whitney U test are displayed in asterisks if <0.05. ∗p value < 0.05, ∗∗p value < 0.01. See also Figures S3, S4, and S5 and Tables S5 and S6.

**Figure S3:**
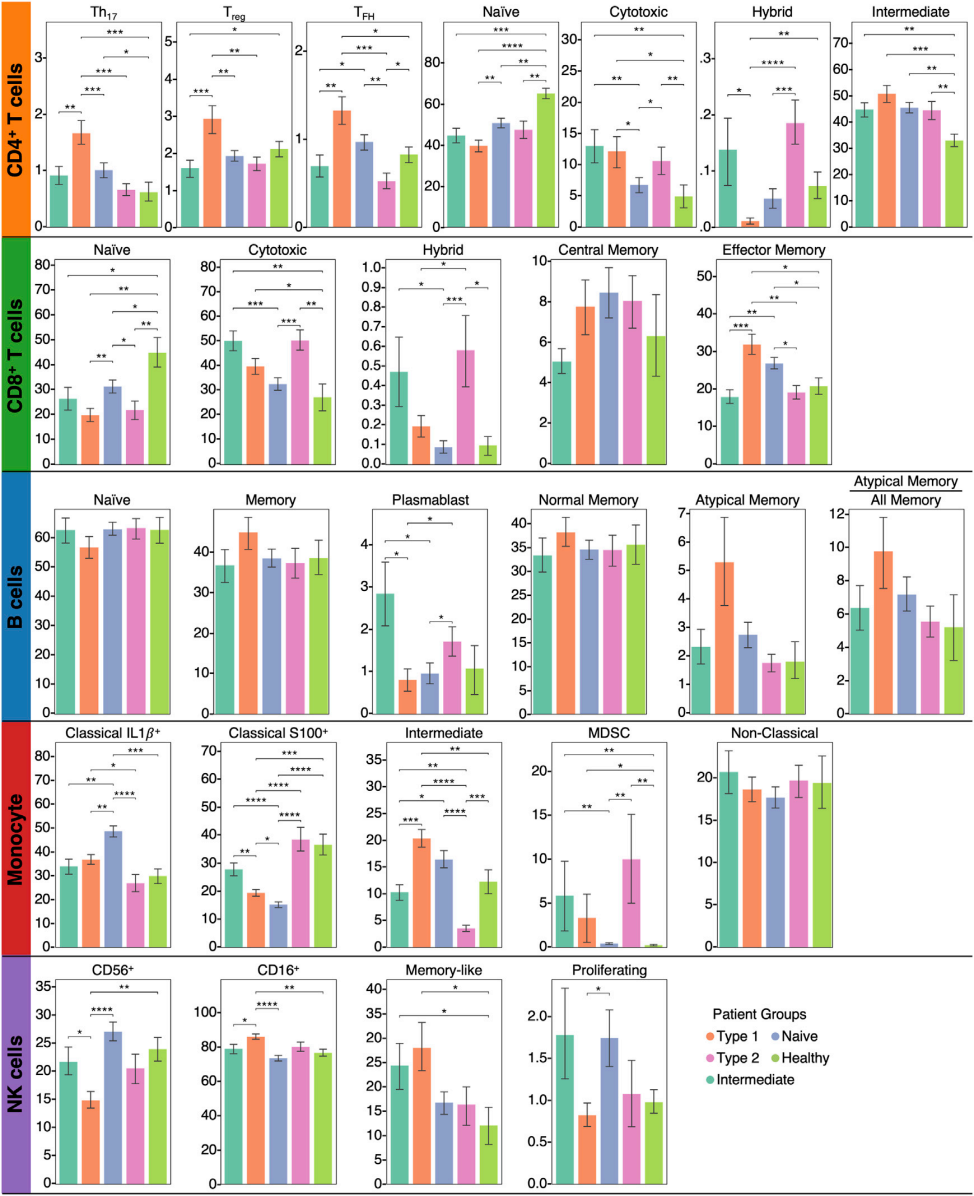
Bar plots showing the percentages of subtypes of CD8^+^, CD4^+^ T cells, B cells, monocytes, and NK cells as measured from 10x data at the convalescent stages for each patient group, related to Figure 5 Data are represented as mean ± SE. p values calculated from the Mann-Whitney U test are displayed in asterisks if <0.05. ∗p value < 0.05, ∗∗p value < 0.01, ∗∗∗p value < 0.001, and ∗∗∗∗p value < 0.0001.

**Figure S4:**
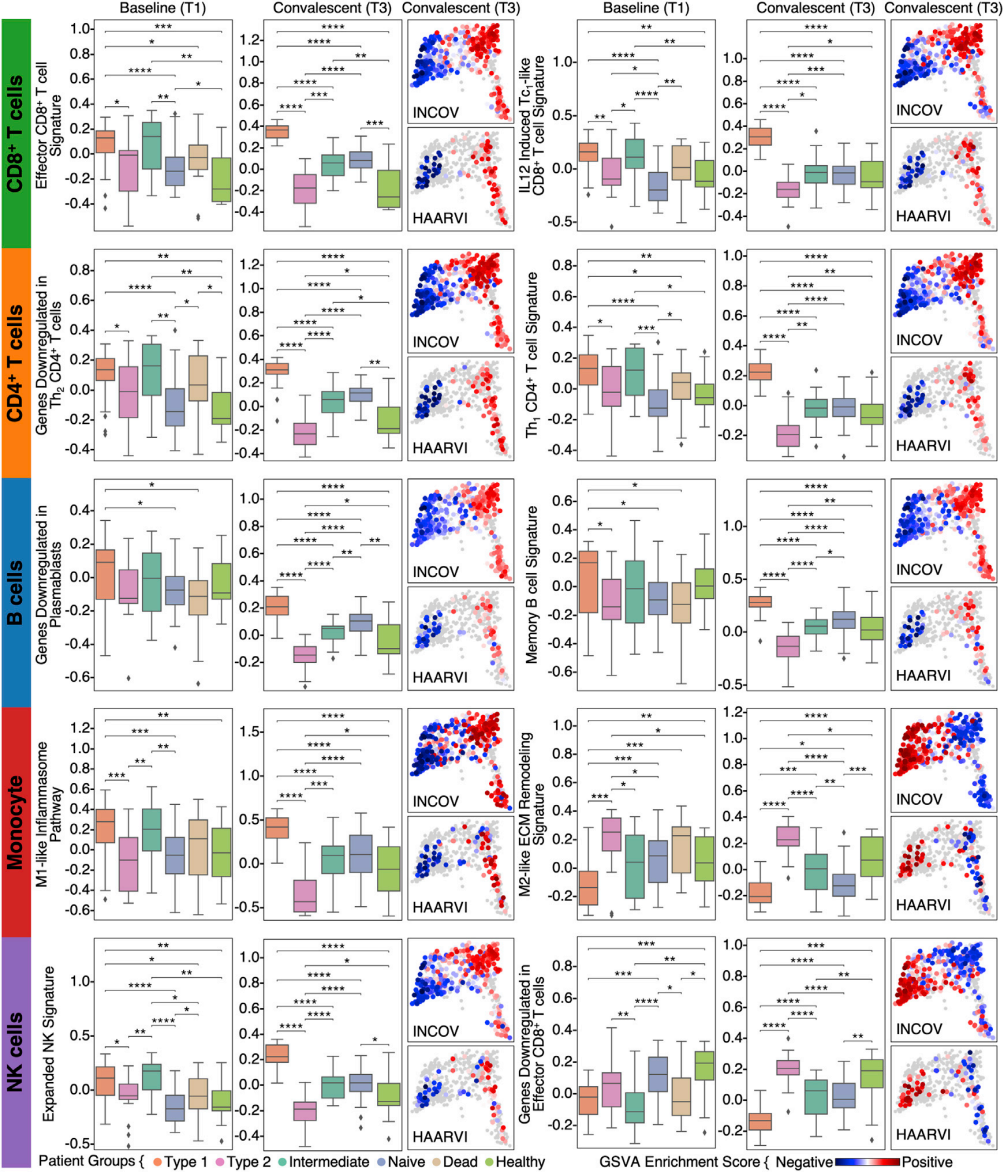
Pathway analysis of patient-group-specific transcriptomic signatures in CD8^+^ T and CD4^+^ T cell, monocytes, and B cells across time and patient cohorts, related to Figure 5 Two pathways are shown for each cell types. Left two boxplots for each pathway indicate the enrichment score of a specific pathway across the four patient groups at T1 and T3. Unexposed healthy controls and deceased patients are also included as comparisons (see color key at bottom). The right two projections for each pathway color code the pathway-enrichment score for each blood draw onto their respective dots (each dot represents a patient blood draw) on the map of Figure 5B for INCOV (upper) and HAARVI (lower) cohorts. p values calculated from the Mann-Whitney U test are displayed in asterisks if <0.05. ∗p value < 0.05, ∗∗p value < 0.01, ∗∗∗p value < 0.001, and ∗∗∗∗p value < 0.0001.

**Figure S5:**
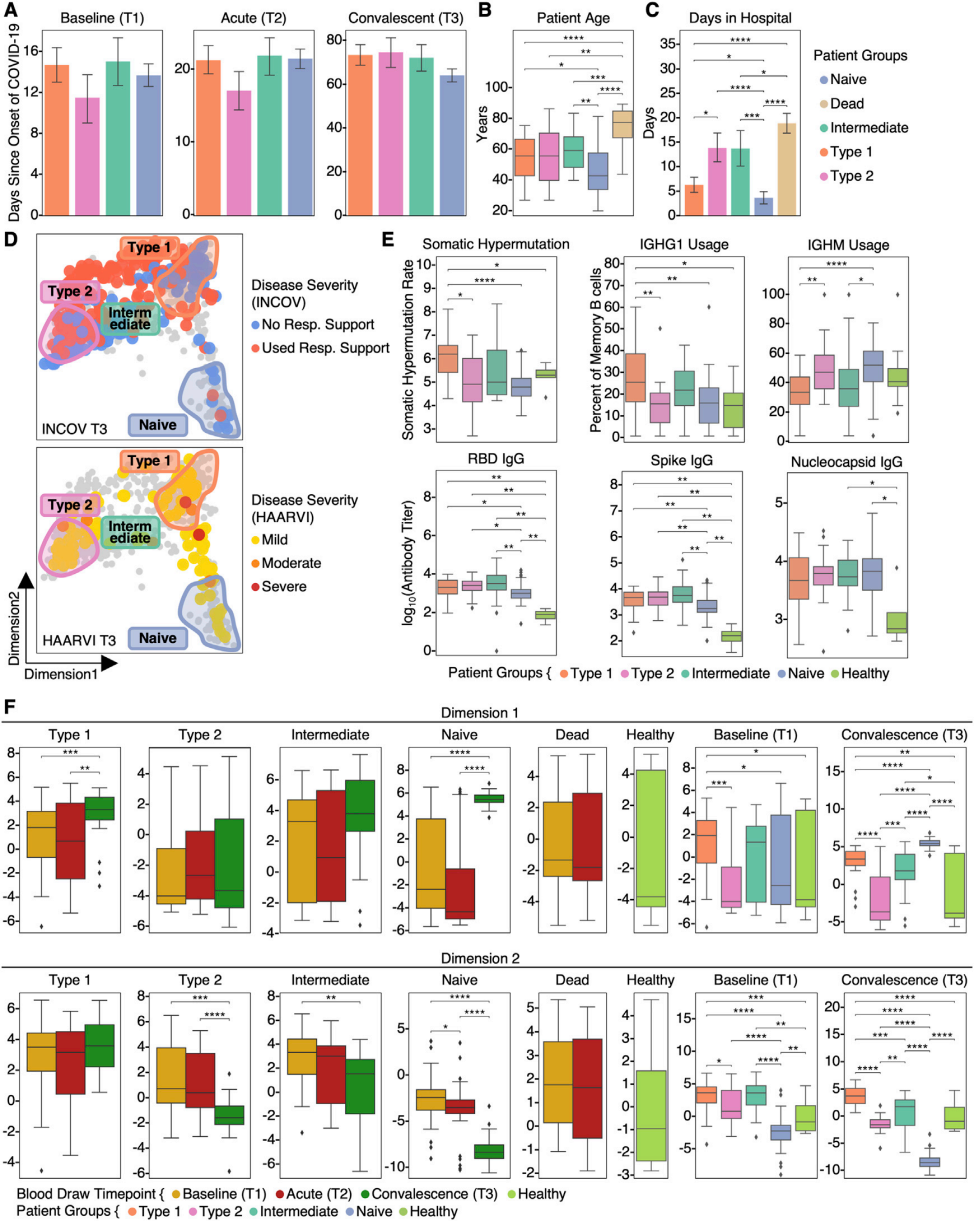
Clinical and functional characteristics of patient groups and dimensional projection validity, related to Figure 5 (A) Bar plots showing the time between onset of COVID-19 to each of the blood draws across four patient groups. Data are represented as mean ± SE. FDR are displayed. No significant differences are seen. (B) Boxplot showing patient age upon enrollment. FDR are displayed. p values calculated from the Mann-Whitney U test then corrected as FDR via the Benjamin-Hochberg method are displayed in asterisks if FDR < 0.05. ∗FDR < 0.05, ∗∗FDR < 0.01, ∗∗∗FDR < 0.001, and ∗∗∗∗FDR < 0.0001. (C) Bar plot showing days in hospitals across patient groups. Data are represented as mean ± SE. FDR are displayed. p values calculated from the Mann-Whitney U test then corrected as FDR via the Benjamini-Hochberg method are displayed in asterisks if FDR < 0.05. ∗FDR < 0.05, ∗∗FDR < 0.01, ∗∗∗FDR < 0.001, and ∗∗∗∗FDR < 0.0001. (D) Dimension reduction visualization from Figure 5 with INCOV and HAARVI cohorts overlayed and colored by their respective definitions and measurements of disease severity. Immune endotypes are circled. (E) Boxplot showing somatic hypermutation (SHM) rates in memory B cells (upper left), percentages of *IGHG1* (upper middle) and *IGHM* (upper right) memory B cell clones over all memory B cell clones, and RBD (lower left), spike (lower middle), and nucleocapsid (lower right) IgG log_10_(titers) for patients at T3. ∗p value < 0.05, ∗∗p value < 0.01, ∗∗∗p value < 0.001, and ∗∗∗∗p value < 0.0001. (F) Boxplot showing the dimension-1 (y axis of top row) and dimension-2 (y axis of bottom row) values of four patient groups controls across time points in comparison with dead patients and unexposed healthy. p values calculated from Mann-Whitney U test are displayed in asterisks if <0.05. ∗p value < 0.05, ∗∗p value < 0.01, ∗∗∗p value < 0.001, and ∗∗∗∗p value < 0.0001.

To systematically investigate the association between PASC and these altered immune states at T3, we studied the sc-RNA-seq data for transcripts enriched for a given PASC. The mean expression of these gene modules comprises symptom-immune signatures, which are visualized in a two-dimensional map to help visualize these signatures (Figures 5A and 5B; STAR Methods). When individual patients, based upon T3 data, were projected onto the map, four patient groupings, or endotypes, were resolved (Figures 5B and S3; Tables S6.1 and S6.8). This same sc-RNA-seq analysis performed on the independent HAARVI cohort revealed a similar immune polarization pattern (Figures S4 and S5D), suggesting that such polarization may be broadly shared across post-acute COVID-19 patient populations.

**Figure S6:**
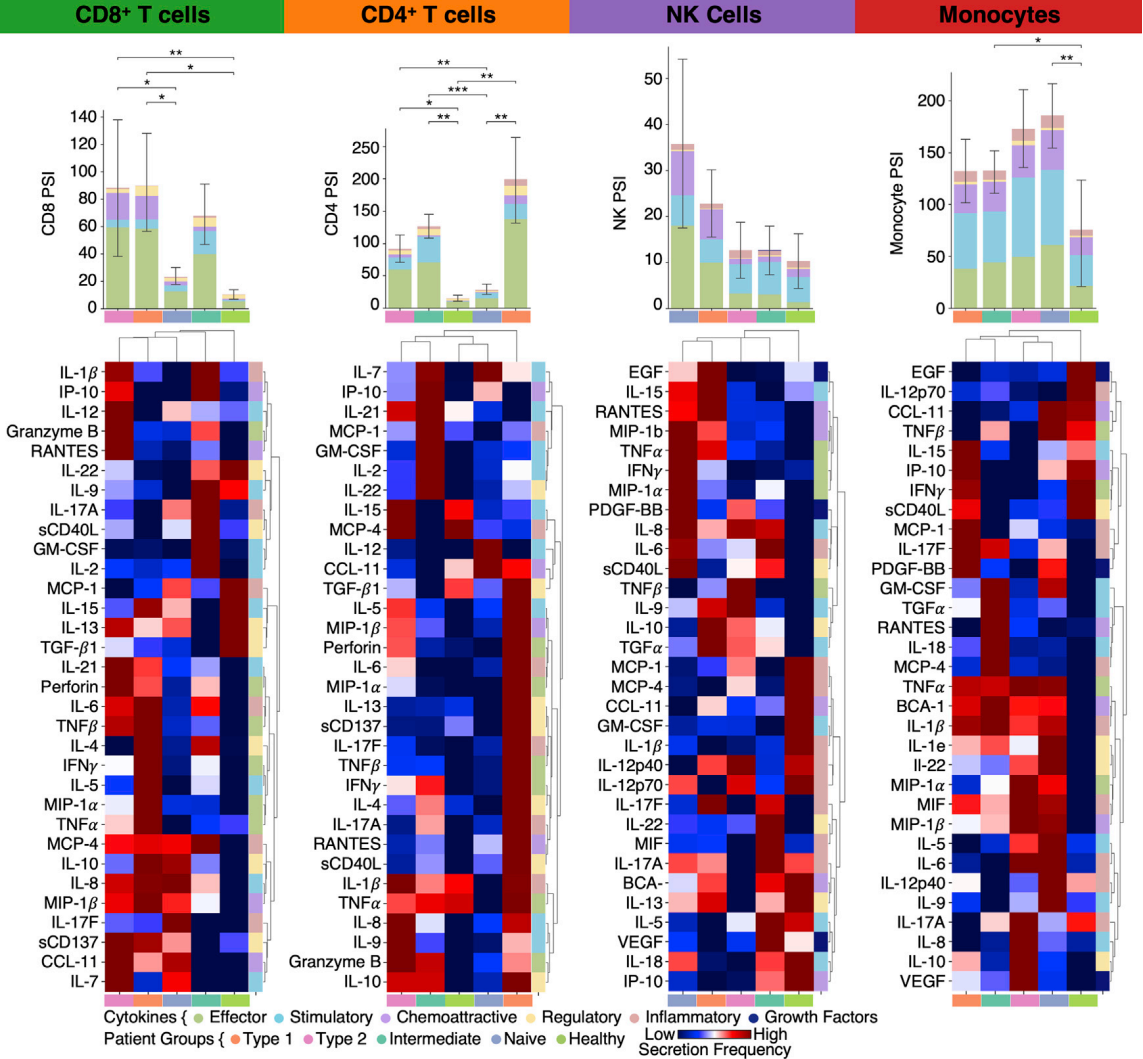
Single-cell secretome functionality analysis and phenotype-PASC association analysis, related to Figure 6 Single-cell secretome analysis of the functionalities in different immune cell types. Top row: single-cell polyfunctional strength index (PSI) of each cell type in each patient group and unexposed healthy control (see color key at the bottom). Data are represented as mean ± SE. Bottom row: heatmap visualization of average cytokine secretion frequencies for each cell type for each patient group at convalescence or healthy unexposed control (see color key at bottom). p values calculated from the Mann-Whitney U test are displayed in asterisks if <0.05. ∗p value < 0.05, ∗∗p value < 0.01, and ∗∗∗p value < 0.001.

**Figure S7:**
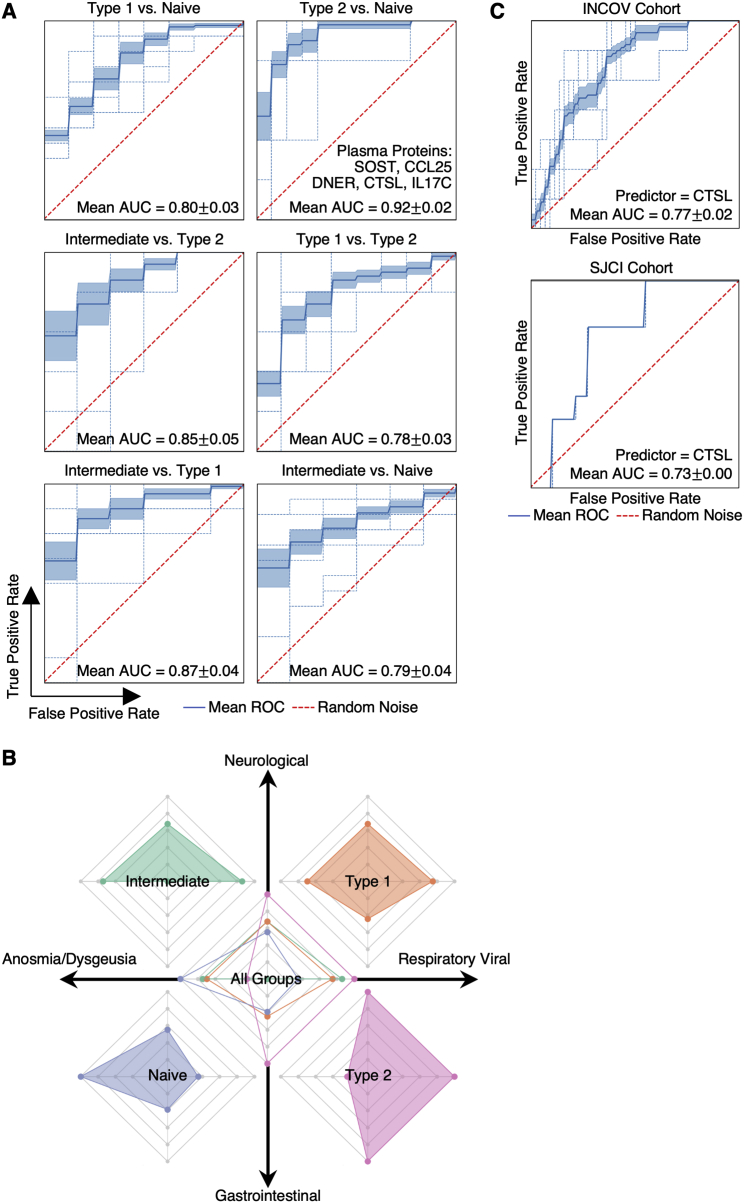
Machine learning model evaluation and multi-omic PASC associations, related to Figure 5 (A) Receiver operating characteristic curves, per cross-validation (CV) iteration, for pair-wise classification (see subtitles) based upon the levels of five markers at T1 for different validation pairs. Area under the curve (AUC) values for different CVs (in different colors) are displayed. (B) The four axes of the radar plot indicate the enrichment score for four sets of PASC at T3 for each immune endotype. (C) Receiver operating characteristic curve for survival prediction based on T1 plasma CTSL levels for the INCOV cohort (upper) and validation of the model trained using the INCOV cohort with T1 plasma CTSL levels from an independent cohort (SJCI) (lower).

Pathway analysis of the four patient groups revealed coordinated expression patterns across innate and adaptive immune cell types (Tables S6.2–S6.6), with polarization reminiscent of the canonical type 1 and type 2 immune responses (Annunziato et al., 2015). Specifically, the type 1 group (orange) was enriched with Th_1_-like signatures in CD4^+^ T cells, M1-like pro-inflammatory signatures in monocytes, cytotoxic effector signatures in CD8^+^ T cells and NK cells, and memory signatures in B cells (Figures 5B, 5C, and S4; Tables S6.2–S6.6). In contrast, the type 2 group (pink) was enriched for Th_2_-like CD4^+^ T cell signatures, M2-like (anti-inflammatory) monocyte signatures, and a plasma B cell signature (Figures 5B, 5C, and S4; Tables S6.2–S6.6). The intermediate group (green) exhibited a transitional immune status between types 1 and type 2. The naive group (blue) exhibited naive-like T and B cell signatures, and resting NK cell signatures (Figures 5B, 5C, and S4; Tables S6.2–S6.6). Notably, there were no significant differences in the duration between the onset of initial COVID-19 symptoms and the blood draws across the four groups (Figure S5A). Although all non-naive-like patient groups exhibited elevated levels of CD8^+^ and CD4^+^ T cell polyfunctionality at T3, all patient groups exhibited high monocyte polyfunctionality relative to healthy controls (Figure S6; Table S6.9). This suggests varying degrees of persistent, primed immune activation across all patient groups at convalescence. Most reported PASC (except anosmia/dysgeusia) were less for the naive group (Figure S7B), whereas the type 2 group experienced a higher hospitalization rate (Figure 5D), potentially reflecting how type 2 immunity is not tailored for viral clearance.

### Immune-polarizations independently associate with viral and autoantibody PASC factors

Projections of individual patient T1 to T3 trajectories onto the map revealed that individual participants tend toward their T3 regions even at the time of COVID-19 diagnosis (Figures 5B and S5F). This suggests that patients may be predisposed toward their post-acute status early in the infection course. This, in turn, suggests a possible relationship between these patient groupings and certain PASC factors that are present at T1. In Figure 5E, we plot the group distributions of patients expressing high levels of IFN-α2 autoAbs, or the percent of patients who tested positive for EBV viremia or RNAemia. While patients that express anti-IFN-a2 levels two standard deviations above baseline do not associate with a specific group, patients that express high anti-IFN-α2 levels (≥4 standard deviations) associate with the intermediate immune group (Figure 5E). Patients with EBV viremia are also associated most strongly with this group, whereas RNAemia is non-specific (Figure 5E). The plots suggest that the intermediate immune state, which is characterized by both pro-inflammatory and type 2 immune signatures, is worth deeper exploration. The plots also support that these PASC factors may only minimally impact COVID-19 recovery in the naive immune group, and that polarization away from the naive endotype may increase the risk for most PASC (except anosmia/dysgeusia) (Figure S7B).

The indication (Figure 5B) that a patient endotype at T3 is anticipated by their T1 status prompted us to investigate the T1-measured plasma proteins that could serve as biomarkers to anticipate patient groupings at T3. To this end, we resolved a five-protein panel (Figure S7A; Table S6.7). One of the members of our panel, CTSL, has been reported as playing a key role in facilitating SARS-CoV-2 infection in humans (Zhao et al., 2021), and when measured at T1, CTSL was also predictive of patient mortality for the INCOV cohort (Figure S7C, upper). This was further validated in an independent cohort from St. John's Cancer Institute (SJCI) (Figure S7C, lower).

### Cross-dataset correlations suggest certain independence of the PASC-associated factors

**Figure 6:**
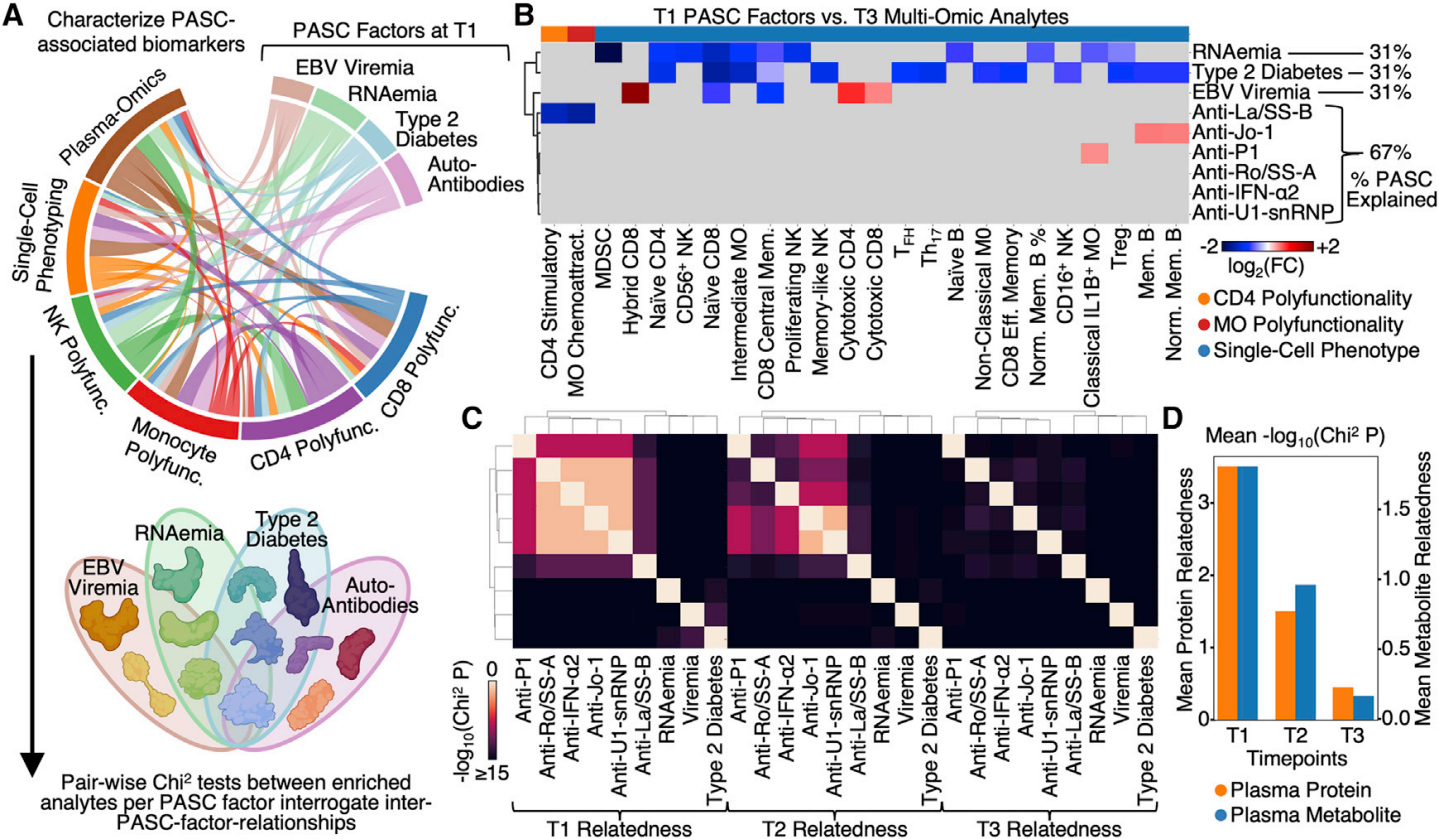
Integrated analysis of associations between multi-omics and PASC factors (A) Illustration of the analysis to identify how the different PASC factors associated with the different multi-omic measurements. (B) Cross-dataset correlations between T1 measurable PASC-associated factors (EBV viremia, RNAemia of SARS-CoV-2, and auto-antibodies) and analytes from different T3 omics (see color key at bottom). Association was quantified via log_2_-fold change values where red indicates positive associations, blue indicates negative association, and gray indicates no significant associations (p ≥ 0.01). (C) Heatmap visualization of the interdependence of the four PASC factors across three time points. The relatedness score represents how significantly the enriched plasma protein sets for each PASC factor overlapped with each other. These are visualized in a pair-wise manner in the matrix. (D) Bar plot illustrating the quantification of the relatedness from (C) plus an analogous analysis for plasma metabolites. The bar heights represent the average non-self pair-wise relatedness value from the heatmaps in (C) with separate y axes for plasma proteins and plasma metabolites. See also Figure S6 and Table S7.

We probed for relationships between the different PASC-anticipating factors and the multi-omic datasets collected at T3. For example, we found that EBV viremia uniquely correlated with percentages of both cytotoxic CD4^+^ and CD8^+^ T cells, as well as proliferative-exhausted (hybrid) CD8^+^ T cells at T3 (Figure 6B; Tables S7.1). However, surprisingly, very few specific multi-omic associations are shared between the PASC factors. This prompted us to probe for relationships (relatedness versus independence) between the T1-measured PASC factors evolve over time (Figure 6C). For this purpose, we queried for plasma analytes that were simultaneously significantly enriched for more than one T1 PASC factor (Figure 6D; Tables S7.2 and S7.3). In fact, several shared relationships are revealed at T1, including cross-associations between all of the autoAbs. These autoAb relations may support the hypothesis suggested by Figure 2 that relates anti-IFN-α2 and ANA autoAbs. By T2, these relationships are diminished, and by T3, the PASC factors appear virtually independent of each other (Figures 6C and 6D). This sharp decrease over time of the relatedness between the PASC factors provides the interesting insight that different T1-measured PASC factors can exhibit similar immunological impacts early in the infection course, but these similarities are rapidly lost over time. This highlights the importance of measurements early in the COVID-19 disease course for understanding these early-time immunological perturbations.

## Discussion

Post-acute sequelae from COVID-19 (PASC) are an emerging global health crisis. We used longitudinal, multi-omic profiling of a few hundred COVID-19 patients and healthy controls to advance the fundamental understanding of the heterogeneity of PASC and to reveal that various PASC-anticipating biological factors (PASC factors) can be measured as early as at initial COVID-19 diagnosis, including pre-existing type 2 diabetes, assessments of SARS-CoV-2 RNAemia, EBV viremia, and autoAbs from the patient blood. Since symptoms can often arise from multiple sources, the identification of discrete and quantifiable PASC factors should be of fundamental importance for understanding PASC and developing treatments. For example, the importance of T1 detectable virus as a PASC factor may suggest that antivirals administered early in the disease course may be beneficial not just for treating acute COVID-19 but also for reducing later PASC. Similarly, the association of cortisol deficiency in patients with respiratory-viral PASC may suggest cortisol replacement therapy as a potential treatment. The association between T2 hyperinflammation with PASC-anticipating autoAbs further implies that therapies controlling hyperinflammation in the acute stage may influence PASC. However, the detailed timing and context of these therapies matter; thus, future well-controlled studies will be needed to test these and other therapeutic implications. The anticorrelations between anti-SARS-CoV-2 IgGs and certain autoAbs may suggest that patients with elevated autoAb levels are more susceptible to breakthrough infections.

The kinetic aspects of this longitudinal investigation were revealed in a number of ways. First, GI PASC uniquely correlates with the newly expanded cytotoxic CD8^+^ and CD4^+^ T cell populations at T3, including SARS-CoV-2-specific clonotypes, which get activated not during acute disease but at convalescence when PASC was identified (Figures 3 and 4). Whether this correlates with the reported GI viral shedding that can occur in some post-acute COVID-19 patients (Parasa et al., 2020) will require additional studies, but the finding that GI PASC also involves bystander activation of CMV-specific T cells (Figures 4B, 4D, and 4E) suggests that additional levels of non-specific T cell activation may also contribute to GI PASC. The activation of auto-reactive T cells has been reported in many infection settings, including COVID-19 (Getts et al., 2014; Woodruff et al., 2020).

A second notable finding from our kinetic analysis is that the participants resolve into one of four immune endotypes at T3, and a participant can be matched with this T3 endotype using measurements at T1. Although this is consistent with the observation that most of the PASC factors can be measured at T1, associations between the PASC factors and the endotypes were only partially resolved. The type 1 and type 2 endotype polarization represent how the immune system evolved to tailor its effector functions to distinct challenges, with type 2 not optimized for viral clearances as reflected by its highest hospitalization rate. Of note is the intermediate “hybrid” group that updates the canonical type 1/type 2 dichotomy of immune polarization. This intermediate endotype warrants further study, as it is associated with patients that exhibit both reactivation of latent EBV as well as patients that express high levels of anti-IFN-α2 autoAbs. Furthermore, the fact that the naive (less-activated/polarized) T3 group exhibited less enrichment for many PASC provides support for the hypothesis that unresolved/persistent immune activation and PASC are associated (Proal and VanElzakker, 2021).

A third kinetic finding that may inform future PASC studies involves the time-evolving inter-relationships (“relatedness”) of the T1-measurable PASC factors. In convalescence (T3), these PASC factors appear largely independent of each other (Figures 6B and 6C, right), which, in the absence of additional data, might suggest that these PASC factors constitute relatively independent treatment targets. However, at diagnosis (T1), these PASC factors exhibit a number of inter-relationships (Figure 6C, left), perhaps suggesting a more limited number of treatments. The implication is that the functional relationships between the various T1 PASC factors will be easier to extract through analysis of biospecimens collected early in the infection course. Future studies of other “long” medical conditions, such as post-treatment Lyme disease (Rebman and Aucott, 2020), “chemobrain” (Nguyen and Ehrlich, 2020), and post-ICU syndrome (Brown et al., 2019), may benefit from our methodologies and findings. The rapid loss over time in measurable inter-relationships between the PASC factors is also reminiscent of many complex dynamical systems that evolve in fashions that are highly sensitive to initial conditions (Olsen and Degn, 1985). For this study, these initial conditions are unique to the individual participants, accentuating the need for acute characterizations of patients to resolve their sources of post-acute sequelae and treatments.

Our analyses provided a framework to understand the heterogeneity of “long COVID” and a rich resource for investigating the biological factors that contribute to PASC, which can potentially be utilized to monitor and guide interventional trials to treat and prevent post-acute COVID-19 symptoms.

### Limitations of the study

Our study focused on PASC at 2–3 months post the onset of COVID-19 and thus cannot discern which patients will develop long-term chronic PASC (Taquet et al., 2021). Furthermore, the establishment of causal links between PASC factors and PASC will likely require model studies in which perturbations can be explored. Although we find that several PASC factors are detectable at initial diagnosis, the development of a predictor of PASC will require data from multiple large, independent studies, and it may also require titers for large panels of autoAbs. An additional limitation is study size. Even in a study comprising a few hundred patients, patients that exhibit both a given PASC factor and a specific symptom may constitute only a small subset, making it difficult to establish specific, robust classifications or predictors. Only 2–3 months post infection blood draw samples were available in our HAARVI cohort, which prevented us from utilizing them for T cell dynamic analyses. Furthermore, our study setup was not suitable to identify genomic factors for PASC, although we are contributing genomic data to support international consortia focused on such studies. Another limitation is associated with the genetic evolution of SARS-CoV-2, which may alter the landscape of PASC experienced by patients but is not addressed here. Finally, our blood processing protocols did not preserve granulocytes; therefore, associations between these immune cells and PASC are unresolved.

## STAR★Methods

### Key resources table

**Table:** 

REAGENT or RESOURCE	SOURCE	IDENTIFIER
**Antibodies**		

Anti-CD3	eBioscience	Cat# 16-0037-85; RRID: AB_468855
Anti-CD28	eBioscience	Cat# 16-0289-85; RRID: AB_468927
Anti-SARS-CoV-2 Spike Antibody	Abcam	Cat# ab273073
Anti- SARS-CoV-2 Nucleocapsid Antibody	Abcam	Cat# ab272852
Anti-Human IgG Conjugated with Peroxidase	Sigma Aldrich	Cat# A6029; RRID: AB_258272
Anti-Human IgA Conjugated with Peroxidase	Sigma Aldrich	Cat# A0295; RRID: AB_257876
Anti-Human IgM Conjugated with Peroxidase	Abcam	Cat# ab97205; RRID: AB_10695942

**Chemicals, peptides, and recombinant proteins**		

RBC lysis buffer	Miltenyi Biotech	Cat# 130-094-183
RPMI 1640 Medium	Gibco	Cat# 11875-093
FBS	Gibco	Cat# 15140-122
Glutamax	Gibco	Cat# 35050061
TotalSeq™-C0251 anti-human Hashtag 1 Antibody	Biolegend	Cat# 394661
TotalSeq™-C0252 anti-human Hashtag 2 Antibody	Biolegend	Cat# 394663
TotalSeq™-C0253 anti-human Hashtag 3 Antibody	Biolegend	Cat# 394665
TotalSeq™-C0254 anti-human Hashtag 4 Antibody	Biolegend	Cat# 394667
TotalSeq™-C0255 anti-human Hashtag 5 Antibody	Biolegend	Cat# 394669
TotalSeq™-C0256 anti-human Hashtag 6 Antibody	Biolegend	Cat# 394671
TotalSeq™-C0257 anti-human Hashtag 7 Antibody	Biolegend	Cat# 394673
TotalSeq™-C0258 anti-human Hashtag 8 Antibody	Biolegend	Cat# 394675
TotalSeq™-C0259 anti-human Hashtag 9 Antibody	Biolegend	Cat# 394677
TotalSeq™-C0260 anti-human Hashtag 10 Antibody	Biolegend	Cat# 394679
TotalSeq™-C Custom Human panel	Biolegend	Cat# 99814
CD8 Microbeads	Miltenyi Biotech	Cat# 130-045-201
CD4 Microbeads	Miltenyi Biotech	Cat# 130-045-101
CD56 Microbeads	Miltenyi Biotech	Cat# 130-050-401
Pan Monocyte Isolation Kit	Miltenyi Biotech	Cat# 130-096-537
IL-2	Biolegend	Cat# 589104
Lipopolysaccharide	Sigma Aldrich	Cat# L2654
PMA	Sigma Aldrich	Cat# 8139
Lonomycin	Sigma Aldrich	Cat# 10634
CryoStor CS-10	Biolife Solutions	Cat# 210102
PBS, 1X	Fisher Scientific	Cat# 21-040-CV
SARS-CoV-2 RBD Protein	Invitrogen	Cat# RP-87678
SARS-CoV-2 Spike Protein	Invitrogen	Cat# RP-87680
SARS-CoV-2 Nucleocapsid Protein	Invitrogen	Cat# RP-87707
TMB Peroxidase Substrate Kit	Seracare	Cat# 5120-0047
IFN-α2	Miltenyi Biotech	Cat# 130-093-874
U1-snRNP	Diarect	Cat# A13000
Ribosomal Phosphoprotein P1	Diarect	Cat# A14200
Ro/SS-A	Diarect	Cat# A12700
La/SS-B	Diarect	Cat# A12800
Jo-1	Diarect	Cat# A12900

**Critical commercial assays**		

Chromium Next GEM Single Cell 5’ Library and Gel Bead Kit v1.1	10x Genomics	Cat# 1000165
Chromium Next GEM Chip G Single Cell Kit	10x Genomics	Cat# 1000120
Single Cell Polyfunctional Strength Panel Human	Isoplexis	Cat# PANEL-1001
Single Cell Polyfunctional Strength ISOCODE Chip	Isoplexis	Cat# ISOCODE-1000
Single Cell Innate Myeloid Panel Human	Isoplexis	Cat# PANEL-3L02
Single Cell Innate and Myeloid ISOCODE Chip	Isoplexis	Cat# ISOCODE-3000
Cardiovascular II panel	Olink	Cat# 95500
Inflammation panel	Olink	Cat# 95302
Metabolism panel	Olink	Cat# 95340
Immune Response panel	Olink	Cat# 95320
Organ Damage panel	Olink	Cat# 95331

**Deposited data**		

Processed scRNA-seq data	This paper	Array Express: E-MTAB-10129

**Software and algorithms**		

Robustbase (Python package)	Maechler et al., 2021	https://github.com/cran/robustbase
CellRanger v3.1.0	10x Genomics	https://support.10xgenomics.com/single-cell-gene-expression/software/pipelines/latest/what-is-cell-ranger
Scrublet v0.2.1 (Python package)	Wolock et al., 2019	https://github.com/AllonKleinLab/scrublet
Scanpy v1.6.0 (Python package)	Wolf et al., 2018	https://github.com/theislab/scanpy
UMAP v0.5.1 (Python package)	McInnes et al., 2020	https://github.com/lmcinnes/umap
Leiden v0.8.0 (Python package)	Traag et al., 2019	https://github.com/vtraag/leidenalg
bbKNN v1.3.12 (Python package)	Polański et al., 2020	https://github.com/Teichlab/bbknn
Scirpy v0.3 (Python package)	Sturm et al., 2020	https://github.com/icbi-lab/scirpy
BLASTp v2.12.0+	Altschul et al., 1997	https://blast.ncbi.nlm.nih.gov/Blast.cgi?PAGE=Proteins
Lifelines v0.26.0 (Python package)	Davidson-Pilon, 2021	https://github.com/CamDavidsonPilon/lifelines
scikit-learn v0.24.2 (Python package)	Pedregosa et al., 2011	https://github.com/scikit-learn/scikit-learn
IgBLAST	Ye et al., 2013	https://www.ncbi.nlm.nih.gov/igblast/
Immunarch v0.6.5 (R package)	ImmunoMind Team, 2019	https://immunarch.com/
IsoSpeak Software	Isoplexis	Product Code: ISOSPEAK-1000-1

**Other**		

IsoLight System	Isoplexis	Product Code: ISOLIGHT-1000-1
EDTA-coated Vacutainer Tubes	BD	Cat# 366643
384-well plates	Thermo Fisher	Cat# 464718

### Resource availability

#### Lead contact

Further information and requests for resources and reagents should be directed to and will be fulfilled by the lead contact, Dr. James R. Heath (jim.heath@isbscience.org).

#### Materials availability

This study did not generate new unique reagents.

### Experimental model and subject details

#### COVID-19 and healthy individuals

The INCOV cohort included 209 SARS-CoV-2 patients (50% females, aged between 18 and 89 years with an average of 56 years), an expansion on the cohort previously published at acute infection (Su et al., 2020). Potential participants were identified at five hospitals of Swedish Medical Center and affiliated clinics located in the Puget Sound region near Seattle, WA. All enrolled patients provided written in-person informed consent. De-identified proteomic and metabolomic data from matched healthy controls processed using the shared technical pooled control samples to enable batch-correction were previously collected from individuals enrolled in a wellness program (Manor et al., 2018) (Arivale, Seattle, WA). Healthy control samples for single-cell analyses were obtained from Bloodworks Northwest (Seattle, WA). Detailed information on age, sex, race, ethnicity, and disease history *etc*. of this patient cohort and healthy controls are listed in Tables S1.1 and S1.2. Disease severity was quantified using the WHO Ordinal Scale for Clinical Improvement score (WOS) (World Health Organization, 2020). Clinical data for hospitalized patients were abstracted from deidentified electronic health records (EHR). Clinical lab data were extracted from the nearest time point to each blood draw. Procedures for the INCOV study were approved by the Institutional Review Board (IRB) at Providence St. Joseph Health with IRB study number STUDY2020000175 and the Western Institutional Review Board (WIRB) with IRB study number 20170658.

The HAARVI (hospitalized or ambulatory adults with respiratory viral infections) cohort included 100 individuals that were either inpatients or outpatients with laboratory-confirmed SARS-CoV-2 infection. The HAARVI participants were aged between 23 and 76 years with an average of 50 years, with 66% females. Inpatients were hospitalized at either Harborview Medical Center, UW Medical Center Montlake, or UW Medical Center Northwest and were enrolled during their hospital admission. Outpatients were identified through a laboratory alert system, email and flyer advertising, and through positive SARS-CoV-2 cases reported by the Seattle Flu Study (Chu et al., 2020). All participants subsequently were asked to return at day 60 or 90 for follow-up. Blood draws were taken during their follow-up visits. Participants or their legally authorized representatives completed informed consent. Demographics are listed in Table S1.4. The HAARVI study was approved by the IRB at University of Washington with IRB study numbers STUDY00000959 and STUDY00002929.

Additionally, plasma samples were obtained from a third cohort SJCI (St. John's Cancer Institute) where SARS-CoV-2 patients were consented under PH&S IRB approved protocol SJCI(JWCI)-18-0401, PH&S IRB number STUDY2018000254. The JWCI/SJCI cohort contained 33 subjects. Participants were aged between 28 and 96 years with a median of 66 years. 36% were female. Large fractions of the cohort had hypertension (67%) and/or diabetes mellitus (36%) among other comorbidities. SARS-CoV-2 severity spanned from 3 to 7 on the WOS (median 5).

### Method details

#### Symptom survey

Persistent symptoms at the T3 draw were determined by implementing interview survey, complemented by a chart review, which were completed on 94 (75%) and 125 (100%) of convalescent patients, respectively. A standardized list of symptoms was generated from symptoms known to be common at acute infection and persisting as PASC (Huang et al., 2021; Logue et al., 2021; Nalbandian et al., 2021), and were asked to the interviewees specifically. These interview symptoms were further validated by a detailed chart review for each participant using a standardized tool by clinicians (J.D.G., W.R.B., M.E.M., R.A.C.) and experienced research coordinators (H.A.A., J.W.). Results from the chart review were used to determine the temporal relationship between reported symptoms and SARS-CoV-2 infection. If symptoms were also reported before COVID-19 due to a preexisting medical condition, it will be coded as unknown (NaN).

The study protocol (developed in March, 2020) allowed for interview questions to be asked to the participant about 8 symptoms: fatigue, cough, dyspnea, sputum production, diarrhea, nausea or vomiting, abdominal pain, and dysgeusia. In addition, the IRB allowed for an open-ended question “Could you tell me about your experience and recovery from COVID-19?” to capture other resolved or persisting patient reported symptoms. Two authors (J.D.G. and H.A.) performed quality control and standardization for all reviews which included clarifying with further review of the EHR, notes from chart review and interview and follow-up questions to the reviewer. PASC symptoms were deemed to be present when reported symptoms from the interview and EHR are consistent.

Symptoms were also grouped as follows: respiratory viral (cough, fatigue, shortness of breath, fever or chills, muscle/body aches, nausea), gastrointestinal (diarrhea, abdominal pain), neurologic (anxiety, blurred vision, depression, memory problems, difficulty concentrating, difficulty sleeping, dizziness, headache), and anosmia/dysgeusia (loss of taste, loss of smell).

In our analysis, a value of 1 in respiratory viral suggests >=2 of respiratory viral PASC, and 0 suggests that no respiratory viral PASCs were reported. For the other three categories of symptoms, a value of 1 suggests >=1 PASC reported, and 0 suggests no PASC reported. For single PASC, we only analyzed the ones that were reported by >10 patients, including fatigue, shortness of breath, cough, inability to exercise, memory problems, difficulty concentrating, sputum, listed by frequency in a descending order.

#### Plasma and PBMC isolation

Plasma and PBMCs from the INCOV cohort were isolated from patient whole blood as previously described (Su et al., 2020). Plasma and PBMC fractions were isolated from patient blood collected in EDTA-coated vacutainer tubes. After centrifuged at 800 x g for 15 min at room temperature, the PBMC layer (which did not include granulocytes (such as neutrophils)) was isolated, counted, and aliquoted at 2.5 million cells/ml in CryoStor CS-10 freeze media. The aliquoted EDTA-plasma and PBMCs were frozen at -80°C. PBMCs were later transferred into liquid nitrogen and stored until use.

Participant samples from the HAARVI cohort were collected in acid citrate dextrose and serum-separating tubes (SST, BD). Whole blood in SST tubes was allowed to clot by incubating for at least 1 hr at room temperature then centrifuged at 700xg for 10 minutes, aliquoted, and stored at -20°C. PBMCs were isolated by density gradient centrifugation using Histopaque (Sigma-Aldrich). After washing, purified PBMC were resuspended in 90% heat-inactivated fetal bovine serum (FBS) (Sigma-Aldrich) with 10% dimethyl sulfoxide (DMSO) (Sigma-Aldrich) cryopreservation media and stored in liquid nitrogen until use. All samples were frozen within 6 hrs of collection time.

#### Single-cell multi-omics assay

Chromium Single Cell Kits (10x Genomics) were utilized to analyze the transcriptome, surface protein levels, TCR, and BCR sequences simultaneously from the same cell. Experiments were performed according to the manufacturer’s instructions. Briefly, cryopreserved PBMCs were thawed and incubated with the 1X red blood cell lysis solution (Miltenyi Biotech) to lyse any remaining red blood cells in the PBMC samples. Cells were stained with cell hashtag antibodies (BioLegend) and TotalSeq-C custom human antibodies (BioLegend). Stained cells were then loaded onto a Chromium Next GEM chip G (10X Genomics). Cells were lysed for reverse transcription and complementary DNA (cDNA) amplification in the Chromium Controller (10X Genomics). The polyadenylated transcripts were reverse-transcribed inside each gel bead-in-emulsion afterward. Full-length cDNA along with cell barcode identifiers were PCR-amplified and sequencing libraries were prepared and normalized. The constructed library was sequenced on the NovaSeq platform (Illumina).

#### SARS-CoV-2 viral load measurements

The miRNeasy kit (Qiagen) was used to isolate RNA from 100 μl of plasma or nasopharyngeal swab samples according to the manufacturer’s instructions. The RNA was eluted from the membrane with either 30 μl or 50 μl of RNAse free water for plasma or nasopharyngeal swab samples respectively. To detect viral sequences, protocol from the CDC was followed (Centers for Disease Control and Prevention, 2020), and primers were obtained from Integrated DNA Technologies (IDT). The qRT-PCR results were performed on a CFX-96 qPCR machine (Bio-Rad). Levels of SARS-CoV-2 RNA and human RNase P transcript were expressed as cycle threshold (CT) value. A CT value < 36 was considered positive. The conversion of Ct value to viral copy number is based on a titration curve generated using synthetic partial viral RNA (Twist) with known copy number as a template for qPCR.

#### CMV and EBV viremia measurements

Real-time quantitative PCR was performed to detect and quantify cytomegalovirus (CMV) and Epstein-Barr virus (EBV). The DNA was extracted from 200 μl of plasma using QIAamp 96 DNA blood kit (Qiagen) and eluted into 100 μl AE buffer (Qiagen). 10 μl of DNA was used for each 30 μl PCR assay. 2x QuantiTect multiplex PCR mix (Qiagen) was used for all PCR assays. The PCR cycling steps were as follows: 1 cycle at 50°C for 2 mins, 1 cycle at 95°C for 15 mins, and 45 cycles of 94°C for 1 min and 60°C for 1 minute. Exo internal control was spiked into each PCR reaction to monitor inhibition. A negative result was accepted only if the internal control was positive with a CT within 3 cycles of the Exo CT of no template controls. A standard curve based on titers of 10, 10^2^, 10^3^, 10^4^, and 10^5^ per 10 μl in duplex was included in each PCR run. A PCR run was rejected if the lowest dilution of 10 did not amplify. Detection of ≥1 copy of virus DNA/reaction (50 copies/mL of plasma) was considered positive.

#### Plasma proteomics and metabolomics

Plasma concentrations of proteins and metabolites were measured as previously described (Su et al., 2020). Batch-corrected proteomic and metabolomic data were further adjusted for age, sex and BMI, as well as their interactions, using a set of robust linear regression models estimated for each protein and metabolite separately using the external control sample of uninfected individuals that were selected using propensity score matching on a number of sociodemographic and comorbidity variables from a larger in-house sample. Models were fitted using the lmrob function from the R package robustbase with the 'KS2014' setting (Maechler et al., 2021). Metabolite values were log_2_ transformed prior to further analyses, while protein abundance values (NPX) were already log_2_ scaled. Batch-corrected plasma protein and metabolite levels were converted into Z-scores using the means and the standard deviations estimated for the residuals in the matched control samples, which included corrections for age, sex, and body mass index (BMI).

#### Single-cell multiplex secretome assay

Cryopreserved PBMCs were thawed and incubated in complete medium (RPMI 1640 (Gibco) containing 10% fetal bovine serum (FBS, Gibco), 1x of glutamax (Gibco) and 100U/mL penicillin-streptomycin (Gibco)) overnight at 37°C, 5% CO_2_. After overnight recovery, CD4^+^ and CD8^+^ T cells were isolated using CD4^+^ (Miltenyi Biotec) and CD8^+^ (Miltenyi Biotec) microbeads sequentially. NK cells and Monocytes were isolated using CD56 MicroBeads (Miltenyi Biotec) and the Pan Monocyte Isolation Kit (Miltenyi Biotec), respectively.

The isolated CD4^+^ and CD8^+^ T cells were seeded at a density of 1x10^5^ cells/well in a 96 well-plate and stimulated for 6 hrs with plate-bound anti-CD3 antibodies (eBioscience, pre-coated at 10 μg/ml overnight at 4°C) and 5 μg/mL of soluble anti-CD28 antibodies (eBioscience) in complete medium at 37°C, 5% CO_2_. The isolated NK cells were cultured for 12 hrs in the presence of IL-2 (Biolegend, 10 ng/ml). The enriched monocytes at were seeded at 1x10^5^ cells/mL and stimulated with 10 ng/ml lipopolysaccharide (Sigma-Aldrich) for 12 hrs. After stimulation, the activated cells were collected, washed, and stained with membrane stain (included in the IsoPlexis kit), before being loaded onto the chip consisting of 12,000 chambers pre-coated with an array of 32 cytokine capture antibodies. The NK cells were resuspended in complete RPMI supplemented with PMA (Sigma Aldrich, 5 ng/ml) and Ionomycin (Sigma Aldrich, 500 ng/ml) and then loaded onto the IsoCode chip for the stimulation during the incubation. The chip was inserted into IsoLight for further incubation for 16 hours. Secreted cytokines were detected by a cocktail of detection antibodies followed by the fluorescent labeling. Fluorescent signals were analyzed by the IsoSpeak software to calculate the numbers of cytokine-secreting cells, the intensity level of cytokines, and polyfunctional strength index (PSI). Measured cytokines in each panel are listed as below.

Single-Cell Adaptive Immune cytokine panel including the following subsets of cytokines. Effector: Granzyme B, IFN-γ, MIP-1α, Perforin, TNF-α, and TNF-β; Stimulatory: GM-CSF, IL-2, IL-5, IL-7, IL-8, IL-9, IL-12, IL-15, and IL-21; Chemoattractive: CCL11, IP-10, MIP-1β, and RANTES; Regulatory: IL-4, IL-10, IL-13, IL-22, TGFβ1, sCD137, and sCD40L; Inflammatory: IL-1β, IL-6, IL-17A, IL-17F, MCP-1, and MCP-4.

Single-Cell Innate Immune cytokine panel including the following subsets of cytokines. Effector: IFN-γ, MIP-1α, TNF-α, and TNF-β; Stimulatory: GM-CSF, IL-8, IL-9, IL-15, IL-18, TGF-α, and IL-5; Chemoattractive: CCL11, IP-10, MIP-1β, RANTES, and BCA-1; Regulatory: IL-10, IL-13, IL-22, and sCD40L; Inflammatory: IL-1β, IL-6, IL-12-p40, IL-12, IL-17A, IL-17F, MCP-1, MCP-4, and MIF; Growth Factors: EGF, PDGF-BB, and VEGF.

#### SARS-CoV-2 ELISAs

Briefly, 384-well plates (ThermoFisher) were coated with 10 μL of 5 μg/mL SARS-CoV-2 RBD (Invitrogen), spike (S) (Invitrogen), or nucleocapspid (N) (Invitrogen) protein in 0.1M carbonate buffer (pH9.6) overnight at 4°C. Plates were washed four times with wash buffer (phosphate buffered saline (PBS) containing 0.05% Tween-20) and blocked with blocking buffer (wash buffer with 5% BSA) for 1 hour at room temperature (RT). Wells were incubated with 30 μL heat-inactivated plasma samples from COVID-19 patients at six serial three-fold dilutions, starting from 1:30 in blocking buffer for 1 hour at RT. The anti-S antibody (abcam) and anti-N antibody (abcam) at nine serial three-fold dilutions, starting from 2 μg/mL were used as positive controls. A non-coating well, a non-binding well, and a blank well as negative controls wells were also included on the plate. After washing four times with wash buffer, wells were incubated with peroxidase-conjugated goat anti-human IgG (Sigma Aldrich, 1:1,000 dilution), IgA (Sigma Aldrich, 1:5000 dilution), or IgM (Abcam, 1:1000 dilution) antibodies in blocking buffer for 1 hour at RT. Wells were washed four times again before incubating with 30 μL 3,3’,5,5’-tetramethylbenzidine (TMB) substrate solution (Seracare, 5120-0047). The TMB reaction was stopped after 5 minutes by adding 1M sulfuric acid. The OD at 450nm was measured on a Spectramax Plate Reader. The ELISA antibody titers were defined as the plasma dilutions that result in the middle response of the positive control and calculated by fitting the background-subtracted data to a four-parameter logistic regression model using the R package nplr (Commo and Bot, 2016).

#### Autoantibody ELISAs

Autoantibody measurements were adopted from the protocol described above with a few modifications as below. In brief, 384-well plates were coated with 2 μg/mL of recombinant IFN-α2 (Miltenyi Biotech), U1-small nuclear ribonucleoprotein (U1-snRNP) (Diarect), Ribosomal Phosphoprotein P1 (P1) (Diarect), Ro/SS-A (Diarect), La/SS-B (Diarect), or histidyl-transfer ribonucleic acid synthetase (Jo-1) (Diarect), followed by incubating with 1:50 dilutions of plasma samples in duplicates. End-point OD at 450nm was measured and recorded. Since it is common for healthy people to have detectable anti-nuclear antibody titers (Pisetsky, 2011; Slight-Webb et al., 2016; Tan et al., 1997), two methods were adopted to analyze the autoantibody data. Observation in Figure 2B was made only using datapoints that had a value greater than mean +2 standard deviations of healthy controls. Other observations associated with autoantibodies were made using all datapoints.

#### Neutralization assay

The pseudo-virus neutralization assay was conducted by Monogram Biosciences as previously described (Goldman et al., 2020). Briefly, pseudo-typed SARS-CoV-2 virus expressing spike proteins was generated based the original Wuhan-Hu-1 strain sequences (GenBank: NC_045512.2). Neutralizing antibody titers were measured by incubating nine serial three-fold dilutions of plasma samples with a starting dilution of 1:40 and SARS-CoV-2 pseudo-typed virus at 37°C for 1 hr. HEK-293 cells expressing ACE2 were added to the 96-well plate and incubated for additional 60-80 hrs at 37°C for luminescence measurements. Neutralization titers were calculated as the plasma dilution conferring 50% inhibition (ID50) of pseudo-virus infection, adjusting for background luminescence measured from the SARS-CoV-2 nAb positive control.

#### MIRA assay

The MIRA assay for identifying antigen-specific TCRs was performed as previously described (Klinger et al., 2015; Nolan et al., 2020). Briefly, different MIRA peptides were used to stimulate T cells and antigen-specific T cells were sorted on FACSAria after overnight incubation. Sorted cells were lysed and RNA was extracted for TCRβ sequencing. Peptide-specific TCRβ chain sequences were obtained.

#### Bulk TCR sequencing

High-throughput TCRβ sequencing were performed as previously reported (Carlson et al., 2013; Chapuis et al., 2019; Robins et al., 2009). Briefly, DNA was extracted from T cells and TCR TCRβ CDR3 (complementarity determining region 3) regions were sequenced using the immunoSEQ® Assay (Adaptive Biotechnologies, Seattle, WA), a multiplex PCR-based method that amplifies and characterizes CDR3 rearranged sequences, with a built-in rigorous PCR amplification bias control and quality assurance.

### Quantification and statistical analysis

#### Single-cell sequencing data processing

Droplet-based sequencing data were aligned and quantified via Cell Ranger Single-Cell Software Suite (v3.1.0, 10x Genomics) using GRCh38 as a reference. Cells from each demultiplexed sample were first filtered for cells with ≥200 genes, then filtered based on 1) <10000 unique molecular identifiers (UMI) counts per cell (library size); 2) <2500 detected genes per cell; and 3) proportion of mitochondrial gene counts (mitochondrial gene UMIs/total UMIs)<10%. Doublets were simultaneously identified in sample demultiplexing or using scrublet (Wolock et al., 2019) and removed prior to the aforementioned filtering. After QC-based filtering, a total of 966,013 (154,745) cells for the INCOV (HAARVI) cohort were retained for downstream analysis. Scanpy (Wolf et al., 2018) was used to normalize cells via CPM normalization (UMI count per cell was set to 10^6^) and log1p transformation (natural log of CPM plus one).

#### Single-cell RNA-seq cell type identification

Normalized, ln(CPM+1), whole transcriptome mRNA data from QC-passing single cells were analyzed via PCA (ARPACK). All 50 PCs were used to calculate a neighborhood graph (n_neighbors=15) which was utilized to determine UMAP (McInnes et al., 2020) coordinates and Leiden (unbiased clustering) clusters (Traag et al., 2019). Clusters were assigned cell types based on canonical immune markers and multi-cell-type clusters were separated via additional UMAP and Leiden cluster calculations. Clusters (19,034 cells for INCOV, 477 for HAARVI) that co-expressed markers from multiple cell types were labeled as low-quality or doublets and removed from further analysis. In total, 946,979 (154,268) cells for the INCOV (HAARVI) cohort were deemed high-quality and assigned cell types; these cells did not show noticeable batch-to-batch variation.

Labeled T cells were used to calculate a CD4^+^ score (sum of min-max-scaled normalized levels of *CD4* transcript and CD4 surface protein) and a CD8^+^ T cell score (sum of min-max-scaled normalized levels of *CD8A* and *CD8B* transcripts, and CD8 surface protein). The two scores were min-max-scaled and then projected for manual gating of CD4^+^ and CD8^+^ T cells. T cells with ambiguous scores were classified as “Other T cells”. Other rare cell types were labeled however their frequencies may not be robust due to the cell numbers sampled.

#### Single-cell phenotype identification

Normalized mRNA values for each major immune cell type (B cells, CD4^+^ T cells, CD8^+^ T cells, monocytes and NK cells) were used to construct single cell whole transcriptome matrices. These matrices were then utilized to calculate PCA values (50 PCs). PCs were used for batch-corrected (using sequencing batch) neighborhood graph, bbkNN (Polański et al., 2020) construction then UMAP and Leiden (unbiased clustering) cluster calculations were conducted. Cells were then additionally screened for potential doublets, clusters with high doublet scores as quantified from raw transcriptomes via Scrublet or expressing markers of other major immune cell types were removed. If doublets were removed, PCA and subsequent kNN graph construction, and UMAP and Leiden calculations were redone.

Phenotypes were assigned based on Leiden clusters and expression of marker genes relevant for each major immune cell type. Additional CD4^+^ T cell phenotypes T_FH_, Treg and Th_17_ were assigned if cells contained normalized mRNA levels above 0.0 (determined via bimodal distribution of mRNA levels from a density plot and justified as non-dropout values) for *CXCR5*, *FOXP3*, or *RORC*, respectively, and were not already assigned as a Cytotoxic or Hybrid cell. All reduced dimensions (PCA, neighborhood graph, UMAP) and clusters (Leiden) for all of the single cell RNA-seq data were calculated via Scanpy (Wolf et al., 2018).

#### Single-cell TCR-seq data processing

Droplet-based sequencing data for T cell receptor sequences were aligned and quantified using the Cell Ranger Single-Cell Software Suite (10x Genomics) against the GRCh38 human VDJ reference genome.

#### Single-cell TCR phenotype associations

Filtered annotated contigs for TCRs were analyzed via scirpy (Sturm et al., 2020). Aforementioned contigs were filtered for either CD4^+^ or CD8^+^ T cells (as identified via single cell RNA-seq analysis) and then subject to clonotype definition and clonal expansion analysis utilizing nucleotide sequences. Samples were then concatenated together and merged with gene expression data for simultaneous single cell TCR and RNA data visualization.

Both the integrated CD4^+^ and CD8^+^ T cell datasets were subject to filtering for cells with complete TCR sequences, defined as a detectable TRA and TRB. TCRs were normalized per sample (patient blood draw) by sampling with (without) replacement TCRs of samples with n-TCRs < (≥) median TCRs per sample. Pheno-tags were created by compounding cell phenotype with blood draw timepoint (filtered for acute and convalescent). TCR x pheno-tag matrix was constructed with values as the percent of cells in the given pheno-tag with the given TCR. Only TCRs present in ≥2 pheno-tags were included, and values were normalized to ln(value+1). The matrix was then ordered and clustered in the same manner as the correlation analyses with t set to “5”, as visually ascertained.

#### PASC and pre-existing conditions

Pre-existing conditions and clinical measurements were fitted to a multivariable logistic regression model of PASC, adjusted for age, sex, and disease severity (WOS>3). Clinical labs were extracted from electronic health records (EHR). Missing labs were assumed to be normal given missingness was generally for outpatients who were asymptomatic or had only mild symptoms with COVID-19. The median values of the normal lab reference range for adults from American Board of Internal Medicine were used to impute missing labs. Clinical measurements with more than 20% missingness were excluded, then imputation was done using k-nearest neighbors (kNN). A total of 113 clinical measures and labs were available for analysis.

Before fitting logistic regression models, selection for clinical variables was done using extreme gradient boosting (XGBoost)1 using R version 3.6.3 and libraries xgboost (Chen and Guestrin, 2016) (version 1.3.2.1) and caret (Kuhn, 2008) (version 6.0-86). XGBoost models were built to predict a binary PASC group, where a value of 1 suggests at least one PASC group reported (respiratory viral, neurologic, and anosmia/dysgeusia) and 0 suggests no PASC group reported. Data was split into training (80%) and test (20%) sets and upsampling was done using caret to balance the training set. Model training was done using 5-fold cross-validation, and model performances were evaluated in the test set. An XGBoost model with 16 clinical measurements and labs had the highest AUC and accuracy on the test set (AUC = 0.788, 95%CI = 0.546 - 1; accuracy = 0.786, 95%CI = 0.492 - 0.953). The 16 clinical variables combined with preexisting conditions and demographics, were then used to build logistic regression models to evaluate their associations with each of the four PASC categories and single PASCs that were reported by > 10 patients.

#### Plasma-omic enrichment in PASC

For plasma proteomic analysis, top differentially expressed proteins (p-values < 5x10^-3^ in t-tests) in patients reported with a grouped PASC compared to those without were subject to Gene Ontology (GO) analysis. The only two biological process GO terms enriched for the top differential plasma proteins associated with neurological PASC are GO:0042321 (negative regulation of circadian sleep/wake cycle, sleep) and GO:0045188 (regulation of circadian sleep/wake cycle, non-REM sleep). The mean of the two plasma proteins (GHRL, ADA) that are associated with these two GO terms were used to plot Figure 1E left panel. For plasma metabolomic analysis, cortisol and cortisone were in the top three differential metabolites in patients reporting respiratory viral PASC compared to those without were selected to plot Figure 1E middle and right panels (Tables S2.1 and S2.2).

#### PASC and viral load measurements

We performed logistic regression of PASC on binary viral load measurements, while adjusting for age, sex, and disease severity. Separate models were fitted for each viral load measurement at each time point to predict the major symptom groups (respiratory viral, neurological, gastrointestinal, and loss of sense) and symptoms reported in at least 10 patients. For EBV, samples with copies per mL greater than 50 were labeled as positive. For SARS-CoV-2 RNAemia and nasal-swab viral load measurements, samples with CT < 36 were labeled as positive. Disease severity at each timepoint was binarized by WOS>3, which characterizes hospitalized patients with respiratory support, as well as ICU admission. Estimates and their 95% Confidence Interval from multiple models were plotted using Python. Extreme estimates with p≈1 were omitted from visualization. Results from EBV viremia measurements at T2 and T3, or nasal-swab viral at T3 were removed from visualizations in Figures S1F and S1G because <10 patients exhibit positive signal at the time specified above, and so conclusions are hard to draw.

#### Antibody and PASC correlation analysis

We applied two methods for analyzing the correlations between antibodies and PASC. In the first method, the magnitude of correlations (displayed in Figures 2C and S2D) was quantified via the log_2_ fold change (fc) of mean antibody levels in patients with a specific PASC to the mean of those without. The fc values were used for plotting the heatmap. Statistical significance of the correlation between an antibody and a PASC was calculated using the Mann-Whitney U test. The second method involved logistic regression for modeling PASC using antibody levels, sex, age, and disease severity as covariates (Figure S2F). Coefficients (ln(odds ratio)) and p values derived from the logistic were used for plotting the heatmaps and annotating the statistical significance. Both methods used antibody levels as continuous variables.

#### Autoantibody and B cell transcriptomics

Each transcript of each b cell type was tested for relationships with autoantibodies using log_2_ fold changes (auto^high^ (>=4 σ +healthy) vs. auto^-^ (<2 σ +healthy)) as quantification of magnitude and Mann Whitney U test as quantification of significance. The threshold of significance was determined as p < 0.05. Analytes that were representative of enriched pathways/functions were selected. A full table of associations between atypical memory B cell transcriptome and autoantibodies is available in Table S2.5.

#### TCR clonal trajectory analysis

For CD8^+^ T cells, TCR groups presented in Figure 3B were hyper-clustered with t set to “5” to ascertain finer resolution of TCR clonotype clusters (Table S3.5). Clonotype clusters are discussed in text and full TCR group assignment for the analyses are provided (Table S3.5). Differential analysis was performed via scanpy.tl.rank_genes_groups (method=“wilcoxon”, n_genes=300) on single cells comparing clonally deleted vs. expanded cells, full differential gene lists are provided (Tables S3.1 and S3.2).

For CD4^+^ T cells, TCR groups presented in Figure 3B were hyper-clustered with t set to “5” to ascertain finer resolution of TCR clonotype clusters (Table S3.6). Clonotype clusters are discussed in text and full TCR group assignment for the analyses are provided (Table S3.6). Differential analysis was performed via scanpy.tl.rank_genes_groups (method=“wilcoxon”, n_genes=300) on single cells comparing clonally deleted vs. expanded cells, full differential gene lists are provided (Tables S3.3 and S3.4).

#### Combining CD8^+^ transcriptomes and TCR targets

Single cell analysis was performed on combined CD8^+^ T cells from INCOV and HAARVI using Scanpy (Wolf et al., 2018). Additional CD8^+^ T cells derived from healthy samples were extracted from published datasets based on previous cell type annotation (Ren et al., 2021; Stephenson et al., 2021). After T cell receptor annotation using Scirpy (Sturm et al., 2020), we removed cells without TCRβ detected. Each dataset was normalized to counts per million and ln + 1 transformed before computing principal component analysis on the combined count matrix. Batch correction was performed by constructing a batch balanced k nearest neighbors (BBKNN) graph across datasets using the first 50 principal components (annoy neighbor approximation, method=umap, metric=angular, k=12, trim=120) (Polański et al., 2020). T cell clusters were computed via Leiden clustering (res=2.1) on the BBKNN graph and annotated by markers for each phenotype: Naïve (SELL, LEF1, CCR7^high^), Central Memory (SELL, TCF7, CCR7_low_), Effector Memory (*GZMK*), Cytotoxic (*GZMB*, *PRF1*), and Hybrid (*GZMK*, *GZMB*, *PRF1*).

SARS-CoV-2-specific TCRs recognizing MHC class I peptides were obtained from ImmuneCODE MIRA dataset (release 002.1) (Nolan et al., 2020) and seven unpublished MIRA experiments. TCRs specific to Cytomegalovirus or Epstein-Barr virus were obtained from VDJdb (release 2021-02-02) (Bagaev et al., 2020). Additional immunosequencing signature of CMV-associated TCRβs were included (Emerson et al., 2017). Single cells were annotated as virus-specific based on matching TCRβ bio identity, defined by CDR3 amino acid sequences, V gene, and J gene. Sample frequency of TCRs (bio identities) per T cell phenotype were calculated and aggregated based on virus specificity to obtain total frequencies of virus-specific T cells per phenotype.

#### BLASTP analysis

From VDJdb (Bagaev et al., 2020), we obtained peptide sequences of antigens targeted by CMV-specific TCRs detected in our sc-CITE-seq dataset. These peptides sequences were compared with non-redundant protein sequences of the SARS-CoV-2 proteome (taxid:2697049) using blastp online web interface (Altschul et al., 1997).

#### PASC and CD8^+^ T cell transcriptomes

PASC associations were quantified by isolating identified SARS-CoV-2 specific CD8^+^ T cells and taking the mean expression of these single cells per patient blood draw. For each symptom group (e.g. GI) six T cell phenotype marker genes were interrogated by taking the mean expression of patients in the symptom group and subtracting the mean expression of patients not in the symptom group. This results in a gene by symptom group matrix where the value is the aforementioned difference value (positive values mean higher in those in the given symptom group, negative values mean higher in those not in the given symptom group).

#### PASC and phenotype percentages over time

Single-cell phenotype percentages were quantified from 10X-omic data where phenotypes were defined in the aforementioned paragraphs regarding sc-CITE-seq analysis. Associations between these percentages and PASC (including grouped PASCs as well as individual PASC that were reported by more than 10 patients) were quantified via log_2_ fold change between those with a given PASC variable compared to those without. Statistical significance were determined by pair-wise (meaning a single phenotype and a single given PASC) Mann Whitney U tests, with significant associations as p < 0.05.

#### Survival analysis of RNAemia

The lifelines package (Davidson-Pilon, 2021) was used to plot Kaplan-Meier (KM) curves for patient survival probability. Date of death was measured as days since onset of initial COVID-19 symptoms. Date of death is irrelevant for survived patients and KM curves were plotted for up to six-months to display all dead patients. Patients were subsetted for those who RNAemia was tested for and further split into those with and without positive RNAemia at T1. These two separate groups of patients were utilized to compute KM curves. Statistics for survival analysis were gathered via a chi-squared test as implemented via scipy.stats.chi2_contingency. We first generated a subset of patients for those who RNAemia at T1 was quantified, same data was used for the survival curve. We then used this subset of patients to calculate a contingency table with rows as RNAemia positive and negative and columns as survived and died. This contingency table was then inputted into the chi2_contingency method from scipy.stats to generate a p-value.

#### Symptom immune-transcriptome association

Symptoms that were universally queried for from INCOV patients (abdominal pain, cough, diarrhea, fatigue, loss of taste, nausea, shortness of breath, and sputum) were interrogated for immune-transcriptome associations through statistical testing using the Mann-Whitney U test with T3 cell type-specific gene expression. Each symptom was assigned a cell-type specific upregulation and downregulation Z-like score by computing the mean expression of their significantly associated (p<0.05) set of genes per patient blood draw subtracting the mean expression of the patient blood draw across all samples (to account for technical bias) and dividing it by the standard deviation of the patient blood draw as determined via all expressed genes (to account for technical variability). These scores were computed for each patient blood draw for both the INCOV and HAARVI cohorts. PCA was computed on the INCOV cohort using the patient blood draw by signature matrix and HAARVI samples were projected onto this PCA space using the INCOV-derived PCA weights. PCs from both cohorts were utilized to calculate a kNN graph and then diffusion map using Scanpy.

The same patient blood draw by signature matrix was filtered for T3 INCOV blood draws which were used to cluster INCOV patients via consensus clustering. This consisted of 1000 iterations where in each iteration a random subset of the features (25%) was used to cluster patients into four groups (via "Wards" algorithm and scipy.cluster.hierarchy’s fcluster method with criterion “maxclust” and t set to “4”). An affinity matrix was constructed for patients where each value was the percent of iterations of the 1000 iterations in which the two patients appeared in the same cluster. This affinity matrix was then clustered using “Wards” algorithm and split into four groups (same method as the clustering done per iteration).

#### PASC factor relatedness and independence

Relatedness was measured via the -log_10_ of the p-value as ascertained from chi-squared (Chi^2^) test as implemented via scipy.stats.chi2_contingency. We first identified plasma-omic sets (one set for plasma proteins and another for metabolites) that were significantly (p < 0.01) enriched for a given PASC factor for each of the three timepoints. We then created contingency table between two PASC factors where the two categories are non-significant and significant. For example, the double positive region would be the number of analytes that the two PASC factors both showed significant enrichment with the same sign for, and the double negative region would be the number of analytes that the two PASC factors both had showed non-significant enrichment. Only relatedness values were utilized. Pair-wise Chi^2^ tests were utilized to display the relatedness heatmaps shown in Figure 6C. Mean relatedness based on pair-wise tests where the two analytes were not the same (i.e. not type 2 diabetes with type 2 diabetes) were plotted as bars in Figure 6D for each plasma-omic.

#### PASC factor ranking analysis

The percent PASC explained is equivalent to the number of patients that have a given PASC factor out of patients with three or more symptoms, considering symptoms as defined in the aforementioned methods where more than 10 patients reported the given symptom.

#### Machine learning for patient group prediction

Z-scores of plasma protein abundance at diagnosis (T1) were used to construct binary logistic regression classifier to predict patient group assignment at T3 using the scikit-learn package (Pedregosa et al., 2011). Analytes were initially filtered for the top n=15 markers based on the average feature weight. The use of n=15 was determined via an elbow plot based method of ranking against weight factor. This was quantified by fitting an ExtraTreesClassifier on 75% of patients and querying for feature importance of each plasma protein marker. Marker robustness was confirmed by repeating this analysis for 1000 iterations via sklearn’s StratifiedShuffleSplit cross-validation object. The top 15 markers that performed well across all iterations and all combos (measured via the mean feature importance) were selected to test five-marker combinations of plasma protein markers.

Each five-marker combination was cross-validated via 10 iterations (using the cross-validation object StratifiedShuffleSplit) with a train size of 75% and test size of 25%. Models were instantiated with a random state of 0 and selected using GridSearchCV which optimize the C parameter from 10^-2^ to 10^13^ on a log scale. GridSearchCv also used a 10 fold cross-validation StratifiedShuffleSplit object (stratification via true patient group assignment). AUC scores were quantified via sklearn’s roc_curve and auc methods.

#### Machine learning for survival prediction

The five-markers used to predict T3 patient group assignment were split into one and two marker combinations and interrogated in the same manner as the five-marker combinations for patient groups in “Machine learning for patient group prediction” with death or no death as the labels. An independent cohort of patients (SJCI) was used to validate survival predictions by taking the same set of cross-validated models (the 10 logistic regression classifiers trained via the 10 subsets of INCOV data) and scoring the entire SJCI cohort. Average ROC and standard error were plotted in the same manner as well for both the INCOV and SJCI cohort.

#### Single-cell BCR & RNA-seq integration

Annotations from sc-RNA-seq were used to define B cell subtypes in the sc-BCR data. Somatic hypermutation rates (SHM) were defined as the percentages of gaps and mismatches in the variable region of the query contig sequence compared to the top germline V gene hit identified through IgBLAST (Ye et al., 2013). Filtered contig outputs from the 10x Genomics Cell Ranger pipeline were used as input to the R package Immunarch (ImmunoMind Team, 2019) to assign clonotypes to memory B cells for each T3 blood draw for calculation of isotype usage in Figure S5.

## Data Availability

• All PBMC sc-RNA-seq data used in this study can be accessed by Array Express under the accession number: E-MTAB-10129. Additional Supplemental Items are available at Mendeley Data: https://doi.org/10.17632/96v329bg7g.1. • This paper does not report original code. • Any additional information required to reanalyze the data reported in this work paper is available from the lead contact upon request.

## Supplemental information

Table S1. Demographics, clinical characteristics, and PASC information on INCOV, HAARVI, healthy controls, and seronegative INCOV patients, related to Figures 1 and S1 Information on basic demographics, clinical characteristics, medical history, and assays performed on each sample are provided. PASC data are also provided in this table.

Table S2. Plasma proteomic, metabolomic, and viral load data, related to Figures 1, 2, S1, and S2 The batch-corrected *Z* score proteomics and metabolomics data adjusted for age, sex, and BMI are included. Plasma viral load of SARS-CoV-2, CMV, and EBV of each blood draw are provided. Statistics related to Figures 2 and S2 are also provided.

Table S3. Differential expression of clonally expanded versus contracted T cells and TCR cluster sequences, related to Figure 3 Differentially expressed genes in CD8^+^ T and CD4^+^ T cells that clonally expanded compared with those clonally contracted. TCR alpha and beta chains from CD8^+^ and CD4^+^ T cells used for lineage-tracing transcriptomic and percentage-based analyses with respective clusterings denoted are also provided. FDR tables related to Figure 3 are also provided.

Table S4. SARS-CoV-2 and CMV antigen-specific TCR analysis, related to Figure 4 Overlap of SARS-CoV-2-specific MIRA TCRs with sc-CITE-seq datasets, BLASTp alignment comparing the targeted CMV antigens with SARS-CoV-2 proteome, sample frequencies of SARS-CoV-2- and CMV-specific T cell subtypes are provided. FDR tables related to Figure 4 are also provided.

Table S5. Cell phenotype percentages and PASC transcriptomic temporal disparity analysis, related to Figures 2, 5, S2, and S3 The percentages of each immune subpopulation of B cells, CD4^+^ T cells, CD8^+^ T cells, monocytes, and NK cells for each sample are shown. PASC transcriptomic temporal disparity analysis that includes statistics for all time point comparisons and for all cell types is also provided.

Table S6. Patient grouping defined by immune polarization and enriched GSVA (**Gene Set Variation Analysis**) pathways and plasma proteins, related to Figures 5, S3, S4, and S6 Patient groupings defined by immune polarization in Figure 5 and enriched GSVA pathways and plasma proteins for each of the patient groupings are shown. FDR tables related to Figures S3 and S6 are also provided.

Table S7. Statistical analysis of PASC factors with multi-omic measurements, related to Figure 6 Statistics of relatedness of PASC factors and their associations with cell polyfunctionality, single-cell immuno-phenotyping, and plasma proteomics and metabolomics are shown.
